# The effect of particle size, morphology and support on the formation of palladium hydride in commercial catalysts†
†Electronic supplementary information (ESI) available: Full materials and methods. See DOI: 10.1039/c8sc03766c


**DOI:** 10.1039/c8sc03766c

**Published:** 2018-10-15

**Authors:** Stewart F. Parker, Helen C. Walker, Samantha K. Callear, Elena Grünewald, Tina Petzold, Dorit Wolf, Konrad Möbus, Julian Adam, Stefan D. Wieland, Mónica Jiménez-Ruiz, Peter W. Albers

**Affiliations:** a ISIS Facility , STFC Rutherford Appleton Laboratory , Chilton , Didcot , OX11 0QX , UK . Email: stewart.parker@stfc.ac.uk; b Evonik Resource Efficiency GmbH , Rodenbacher Chaussee 4 , D-63457 Hanau-Wolfgang , Germany; c Institut Laue-Langevin , 71 Avenue des Martyrs , CS 20156 38042 Grenoble Cedex 9 , France; d Evonik Technology and Infrastructure GmbH , Rodenbacher Chaussee 4 , D-63457 Hanau-Wolfgang , Germany . Email: peter.albers@evonik.com

## Abstract

The relative amounts of hydrogen retained by a range of supported palladium catalysts have been investigated by a combination of electron microscopy and spectroscopic techniques, including incoherent inelastic neutron scattering. Contrary to expectation, the hydrogen capacity is not determined solely by the metal particle size, but it is a complex interaction between the particle size and its state of aggregation. The nature of the support is not only integral to the amount of hydrogen held by the catalyst, it also causes a marked difference in the rate of release of stored hydrogen from palladium. It is more difficult to fully dehydrogenate palladium on/in the porous activated carbon than on the non-porous carbon black based catalyst. The type of support also results in differences in the form of the residual hydrogen: whether it is α- or β-hydride phase, subsurface or in the threefold surface site. Our data on the supported catalysts reinforces what has only been seen previously with palladium black and our computational study provides confirmation of the empirical assignments. We also report the first vibrational spectroscopic study of hydrogen adsorbed at the surface of β-PdH and have observed for the first time hydrogen in the on-top site. This has enabled the relative proportion of bulk- to surface-H occupation in calculated model and in industrial nanoparticles to be estimated.

## Abstract 2

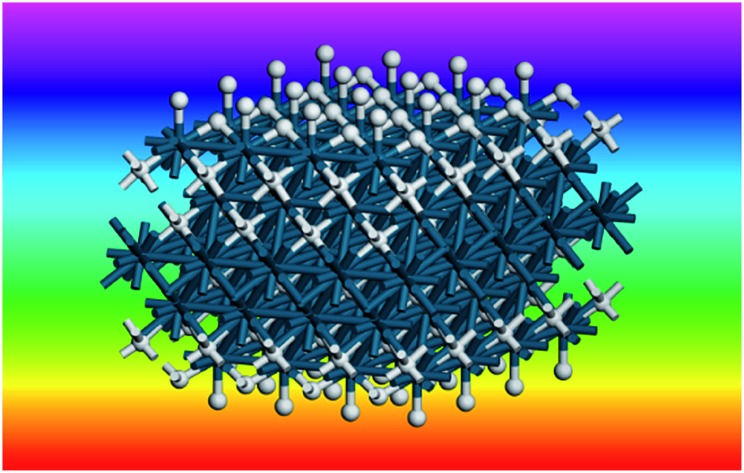
The influence of the support on the quantity of hydrogen present in a supported palladium catalyst is characterised. The on-top hydrogen on β-PdH is observed for the first time.

## Introduction

The ability of palladium metal to reversibly adsorb and desorb large quantities of hydrogen was discovered over 150 years ago.^1^ Since then, this capability has been extensively exploited by the chemical industry in hydrogenation reactions in processes ranging from commodity chemical manufacture to fine chemical production. Examples include: hydrogen peroxide production *via* the anthraquinone process,^2^ the conversion of nitroarenes to anilines^3^ for polyurethane production^4^ and vitamin A synthesis.^5^

In all of these processes it is crucial to control the availability of hydrogen. However, the formation and relative proportion of α- to β-phase palladium hydride, and the width and shape of the two-phase plateau pressure region of the H/Pd-sorption isotherms show variation as a function of primary particle size and size-dependent geometry down to the nanoscale.^6^ Typically, the hydrogen absorption capacity of isolated Pd nanoparticles is lowered, as compared to fine aggregates or to bulk palladium hydride,^7^ while the solubility in the α-phase region is enhanced.^6^,7a Higher hydrogen storage capability at the surface and in sub-surface sites as compared to the bulk has also been reported.^8^

The catalytic activity of palladium depends on the hydrogen concentration: thus β-phase palladium hydride may provide higher activity and lower selectivity in catalytic hydrogenation reactions such as the selective hydrogenation of ethyne than the α-phase hydride.^9^ Alloying or modifying Pd with Pt and Fe in hydrogenation catalysts (*e.g.* for selective hydrogenation of nitroarenes to anilines3a,7c) or controlled poisoning/moderating Pd with Pb in the Lindlar catalyst7*b* has an enormous impact on the hydrogen storage properties of supported palladium entities and are of relevance in balancing catalytic activity and product selectivity.

In a chemical process, dynamic changes in the properties of the working catalyst while passing a loop reactor or operating in a trickle bed reactor,^10^ may occur with changes in the local hydrogen partial pressure or loading. As stated in ref. 11, ‘in many cases a minimum concentration of dissolved hydrogen in the liquid in contact with the solid catalyst is needed’. The choice of solvent and the palladium particle size both influence the turnover frequency, as demonstrated for nitrobenzene hydrogenation over palladium/carbon catalysts.^12^

However, to optimize an industrial hydrogenation process, information on the hydrogenous species present, both on and inside of the supported palladium, is needed. Hence, direct, non-destructive, semi-quantitative measurements of the hydrogen storage and the proton dynamics of nano-sized palladium particles at different degrees of hydrogenation are of great interest. An improved understanding of the varying hydrogen storage and carrier properties in macroscopic, representative quantities of catalyst at realistic hydrogen pressures and with differing precious metal morphologies at the nanoscale are essential.

In the present study, we have characterized a range of commercial catalysts^13^ with different supports and palladium morphology. We have then exploited the high sensitivity of neutron scattering to hydrogen, to observe both surface and bulk palladium hydrides on supported and unsupported palladium catalysts and to semi-quantify the hydrogen content. In the course of this work, we have observed a new adsorption site of hydrogen on β-PdH.

## Results and discussion

### Carbon supports

**Fig. 1:**
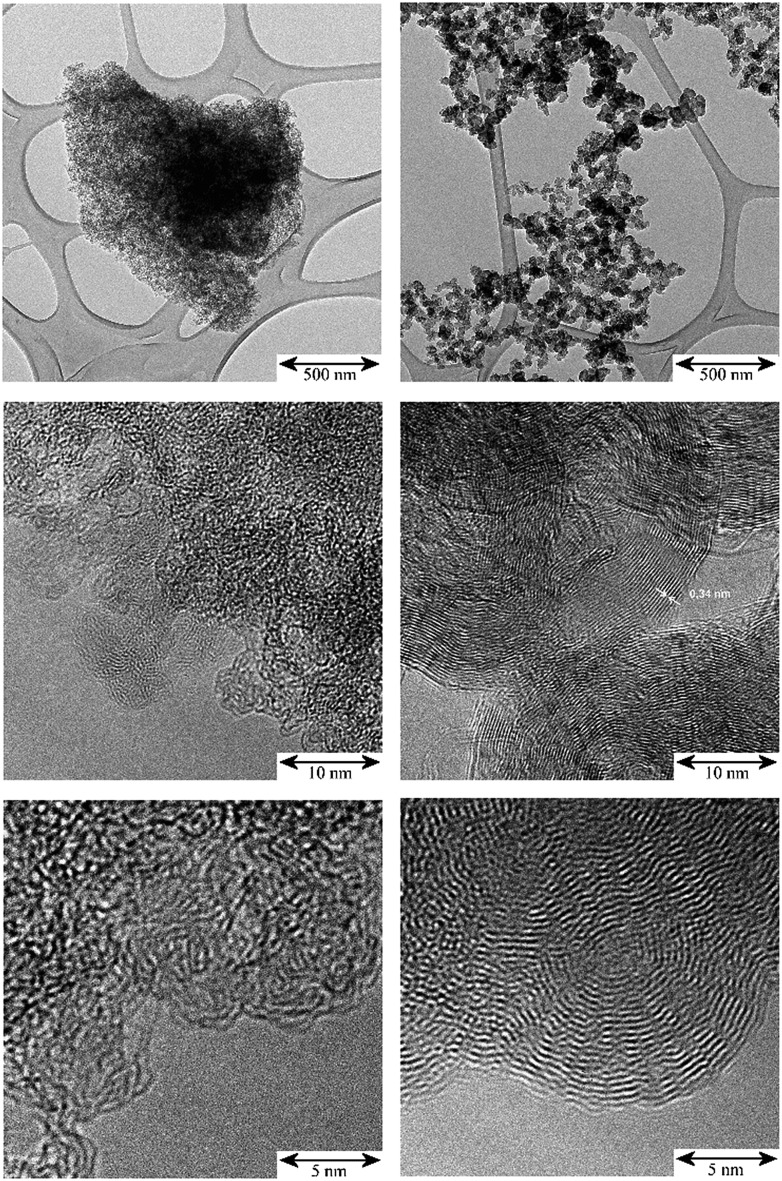
TEM: comparison of the micro- and nano-morphology of the unloaded carbon supports; scale bars from top to bottom: 500 nm, 10 nm, 5 nm. Left: activated carbon (AC); right: carbon black (CB).

The TEM images in Fig. 1 illustrate the different fine structures of the carbon supports as expressed by the very different hydrogen content: 3900 ppm H for the activated carbon (AC) and 295 ppm H for the carbon black (CB). The AC shows the rugged surface of the finely divided powder particles of a *para*-crystalline carbon of moderate sp^2^ character with turbostratic disorder (sp^2^-carbon) and small basic structural units^14^ with enhanced sphericity. The stacking mismatch and enhanced stacking distances (*ca.* 0.37–0.46 nm spacing) indicate the presence of coannular, bent sp^2^-type entities and, potentially, residual traces of disordered sp^3^-type components. The CB shows the characteristic three-dimensional branched aggregate structure of strongly intergrown structural entities generated in the flame synthesis production process and the two-dimensional sp^2^ ordering of graphite layers (0.34 nm) at the surface and in the interior of this non-porous material. At the macroscale, the aggregates show an open structure with high void volume and correspondingly, high macroscopic accessibility for adsorbents. According to the model^15^ of carbon having four different adsorption sites – (I) graphitic planes (sp^2^), (II) amorphous carbon (sp^3^), (III) edges of stacked graphene sheets (sp^2^) and (IV) slit shaped cavities, the TEM images indicate different relative proportions of such sites for the AC and CB. Thus there will be different surface energies for the adsorption properties of these carbons during precious metal impregnation and post-treatment in catalyst manufacture processes.

XPS measurements reveal the presence of chemical differences in the topmost atomic layers. The total oxygen concentration and the ratio of C=O/C–OH-type functional surface groups (observed at *ca.* 531 and 533 eV XPS binding energy values, respectively) was determined as 3.3 at% (ratio *ca.* 0.74) for the AC and 0.3 at% (ratio *ca.* 1.06) for the CB indicating different surface polarity in terms of –OH acidity and, therefore, chemical redox activity in adsorption. For both carbon surfaces, pronounced C1s-plasmon loss features at 291–292 eV of *ca.* 8–10% of the main C1s-signal region indicate a high sp^2^ character of the surfaces.

### Pd-catalysts

**Fig. 2:**
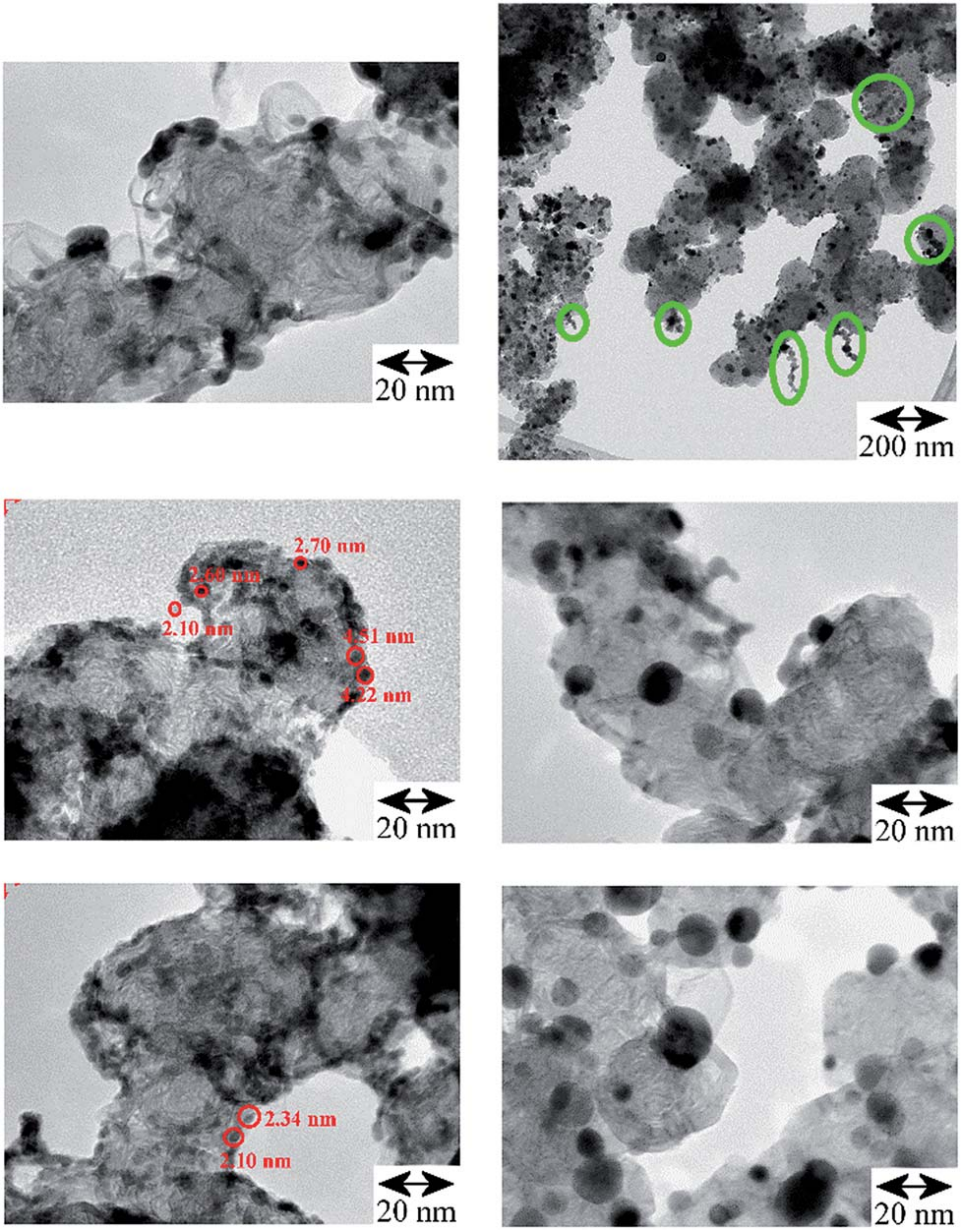
TEM comparison of the Pd/CB catalysts. Left: original catalyst: the linear and branched aggregates are preferentially located at the edges of the CB's support surface, (scale bar 20 nm for all three images; some primary particles are marked with red circles); right, top and middle: after carbo-thermal treatment at 300 °C (scale bar 200 nm and 20 nm) and bottom: 400 °C (scale bar 20 nm), which caused significant particle growth but afterwards aggregates are largely missing and mostly isolated larger Pd crystallites of enhanced average size are left at the CB's surface (Table 1, Fig. S2†). Top right: oval shaped green marks in the survey image of the Pd/CB after 300 °C treatment highlight the presence of residual aggregates, which are absent on the 400 °C catalyst.

TEM images of the Pd catalysts (Table S1†): Pd/AC, Fig. S1† and Pd/CB, Fig. 2, show distinct differences between shape, size and morphology of the precious metal entities. For Pd/AC there are mostly isolated primary particles on/in the porous high surface area activated carbon support. For Pd/CB catalysts, there are mostly linear aggregate chains and branched aggregates, with some agglomerates at the surface. The thermal treatments result in mostly isolated primary particles of enlarged size with only few aggregates or agglomerates remaining.

The results of statistical evaluations of the primary particle sizes are compared in Table 1 (average values) and in Fig. S2† (primary particle size distributions); isolated as well as primary particles forming aggregates were evaluated. The values in Table 1 of DN show that for the Pd-catalysts the average primary particle size is smaller on the CB support than on the AC (2.40 nm *vs.* 3.58 nm) or for PdAu alloy particles on a SiO_2_ support (7.94 nm). The annealing treatments of the Pd/CB catalyst led to significant primary particle growth (2.40 nm → 6.73 nm → 7.74 nm). In parallel the calculated values for the precious metal surface decrease (“TEM-surface” EMS: 186 → 60 → 52 m^2^ g^–1^). The Pd-surface concentrations as measured by means of XPS changed correspondingly: 8.4 → 5.4 → 4.2 at%.

**Table 1:** Results of statistical evaluations of the TEM images and the XPS signal region of the precious metals^*a*^ ^,^^*b*^

Catalyst sample	DN/nm	*S*_DN_/nm	DA/nm	EMS/m^2^ g^–1^	XPS/at%
1, 1.04%Pd.0.43%Au/SiO_2_	7.94	3.54	11.3	44.15	>90 Pd > 90 Au
2, 20% Pd/AC	3.58	2.21	7.44	67.20	15.3
3, 20% Pd/CB	2.40	0.59	2.69	185.84	14.5
4, 3, calcined 300 °C	6.73	2.19	8.27	60.49	27.4
5, 3, calcined 400 °C	7.74	2.53	9.58	52.22	47.9

^*a*^TEM: DN and *S*_DN_: primary particle size (arithmetical average) and its standard deviation; DN = (Σ*n*_*i*_*d*_*i*_)/*N*; DA: primary particle size averaged over the surface, DA = (Σ*n*_*i*_*d*_*i*_^3^)/(Σ*n*_*i*_*d*_*i*_^2^); EMS: calculated surface area by electron microscopy, EMS = 6000/(DA × *ρ*); *ρ*_Pd_ = 12.02 g cm^–3^.

^*b*^XPS: signal contribution of already nearly reduced precious metal species to the whole XPS-signal group, measured in the topmost atomic layers of the freshly impregnated and, for the calcined catalysts and the alloy catalyst, prior to the subsequent *in situ* hydrogenation cycles in the neutron sample cans.

The increasing difference between DN and DA (Table 1) suggests increasing width of the primary particle size distributions and this is compared in more detail in Fig. S2 in the ESI.† Narrow size distribution curves are seen for the Pd/CB and Pd/AC catalysts and shifted, broadened and asymmetric curves after the thermal treatments, indicating that controlled particle growth, surface rearrangement and polydispersity was induced. For the case of alloyed Pd/Au entities supported on the outer perimeter of large spherical and porous SiO_2_ pellets (Fig. S3†), (which was used for comparison of the powder type catalysts with a fixed bed palladium-based catalyst), higher proportions of particles >10 nm are observed (Fig. 2). EDX nano-spot analyses in the FE-TEM (Fig. S3†) confirmed that the supported particles largely consist of a Pd_*x*_Au_*y*_ alloy, with a locally higher Au-level with increasing particle size. For the alloy catalyst's surface the average Pd/Au-ratio measured by XPS was 0.9/0.3 at%.

The TEM images in Fig. 2 show for the Pd/CB catalyst that the thermal treatment led to distinct primary particle growth accompanied by de-aggregation/de-agglomeration of the formerly linear Pd entities. On the freshly impregnated, original catalyst the Pd was deposited as nucleation centers and has grown to small particles at the edges of the graphitic carbon surface (Fig. 1) at “type III” sites to chain-like or branched entities. The thermal treatment induced a rearrangement of the Pd entities. The larger Pd-particles are much more isolated and spherical compared to the smaller chain-like entities. After the 300 °C treatment some residual aggregates could be observed (Fig. 2, top right, marked with circles) which are absent after the 400 °C treatment. Mostly isolated primary particles, however, were already observed for Pd on the high surface area and porous AC powder (Fig. S1,† top left) and also for the Pd/Au alloy particles on the porous SiO_2_ pellets (Fig. S1, bottom, and Fig. S3†).

The XPS results on the relative proportions of reduced precious metal species at the unreduced fresh catalyst's surfaces (Table 1) illustrate, that in the topmost atomic layers of the Pd/CB a thermally induced reduction effect is observed (fresh/300 °C/400 °C: 14.5 → 27.5 → 47.9 at% Pd). This is presumably caused by a removal of OH-groups,^16^ residual Pd-surface/-lattice oxygen and H_2_O species, by partly carbo-thermal reduction and desorption. This may contribute to the de-aggregation/de-agglomeration and improved spreading of the palladium entities over the mostly non-polar sp^2^-surface as with increasing degree of carbo-thermal surface reduction of the precious metal.

### Stored hydrogen

**Fig. 3:**
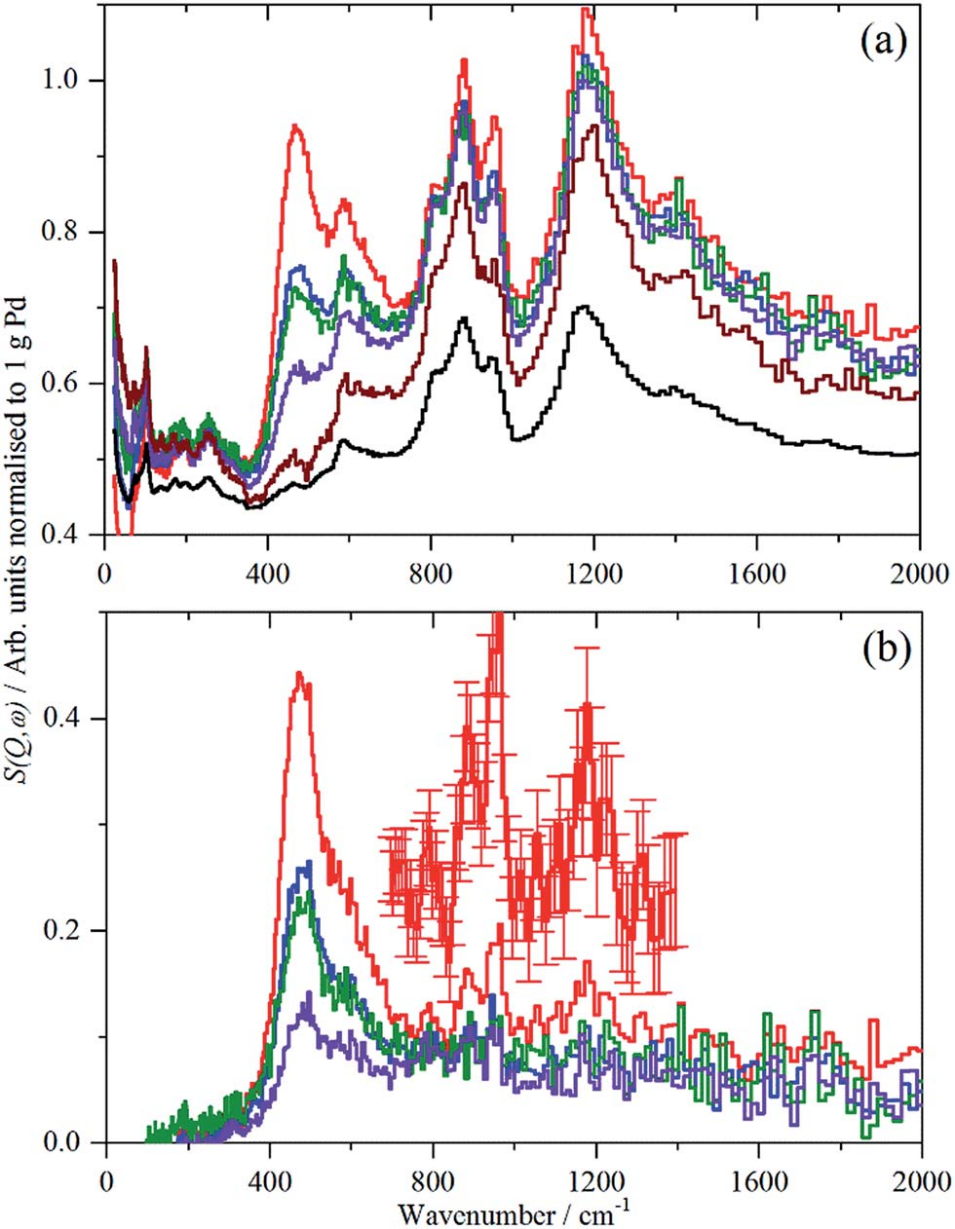
IINS spectra of sample 2, Pd/AC. (a) Normalized spectra as recorded, top-to-bottom: under 700 mbar H_2_, evacuated to 9 mbar H_2_, evacuated to 3 mbar H_2_, evacuated at 200 °C overnight, after second evacuation at 200 °C overnight and the activated carbon support. (b) Difference spectra after subtraction of the sample evacuated for the second time overnight at 200 °C, top-to-bottom: under 700 mbar H_2_, evacuated to 9 mbar H_2_, evacuated to 3 mbar H_2_, evacuated at 200 °C overnight. The inset is *a* × 3 ordinate expansion of the 700–1400 cm^–1^ region of the 700 mbar H_2_ data.

**Fig. 4:**
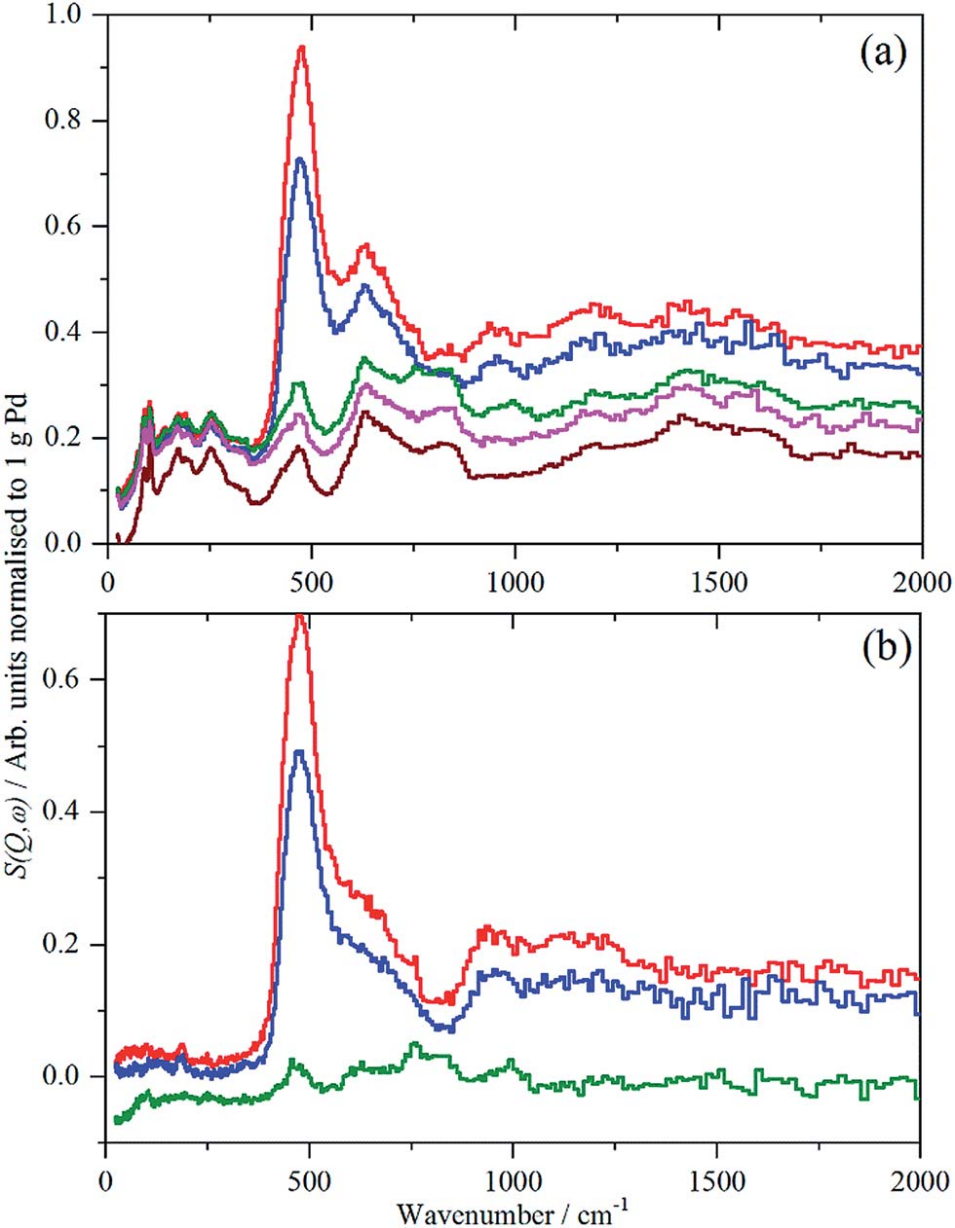
IINS spectra of sample 4, Pd/CB300. (a) Normalized spectra as recorded, top-to-bottom: under 700 mbar H_2_, evacuated to 13.3 mbar H_2_, evacuated to 3 mbar H_2_, evacuated at 200 °C overnight and the carbon black support. (b) Difference spectra after subtraction of the sample evacuated at 200 °C overnight, top-to-bottom: under 700 mbar H_2_, evacuated to 13.3 mbar H_2_, evacuated to 3 mbar H_2_.

For each of samples 2–5, Table S1,† the IINS spectrum was recorded under a saturation pressure of H_2_, (700 mbar), each sample was then progressively dehydrogenated, initially by allowing it to outgas at room temperature for varying times and then by evacuation at 200 °C overnight. Fig. 3 and 4 show the normalized spectra (including that of the carbon support) and the difference spectra for samples 2 (Pd/AC) and 4 (Pd/CB300), generated by subtraction of the sample after overnight desorption at 200 °C. The spectra for samples 3 (Pd/CB) and 5 (Pd/CB400), are very similar to that of sample 4 and are shown in Fig. S4 and S5 (ESI).†

For sample 2, (Pd/AC), the activated carbon support shows the expected^14^ features: 1050–1300 cm^–1^ in-plane C–H bend, 750–900 cm^–1^ out-of-plane C–H bend and 600 cm^–1^ graphene deformations. For samples 3–5, the low hydrogen content (295 ppm H) carbon black support much more resembles graphite.^14^ For all the Pd-containing catalysts, it can be seen that there is an intense, asymmetric peak at 400–600 cm^–1^. This is more clearly seen in the difference spectra, which shows an additional broad feature at 800–1400 cm^–1^ and a weaker feature at 1500–1800 cm^–1^. The three features are assigned6e,^17^,^18^ as the 0 → 1, 0 → 2 and 0 → 3 transitions of β-PdH (vibrational overtones).

This assignment was confirmed by a total neutron scattering study of sample 6 (20% Pd on very low H-content carbon black, 60 ppm H, Table S1†), which showed that at room temperature the sample was actually β-PdH_0.7_, as repeatedly found for nanoparticulate palladium.^6^

The normalized integrated area of the β-PdH feature in each spectrum (determined from the difference spectrum) are summarized in Table 2 (a more detailed version is given in Table S2†). The areas are very dependent on the integration limits (Table S2†), thus the trends are reliable although the absolute numbers are not.

**Table 2:** Integrated hydrogen areas from the IINS spectra in order of increasing average primary particle size DN

Catalyst and dominating morphology^*a*^	DN/nm	IINS area/arb. units normalized to 1 g Pd			
		700 mbar	Degas to 9–14.5 mbar	Degas to 0.1–5 mbar	Extended degas
3, Pd/CB agg	2.40	78.87	55.14	6.84	
2, Pd/AC prim	3.58	59.87	32.97	29.66	12.28
4, 300° prim/agg	6.73	99.00	74.66	18.68	
5, 400° prim	7.74	77.97	23.15	9.06	
1, Pd/Au/SiO_2_ prim	7.94	180			

^*a*^Morphological differences according to TEM: agg = mostly aggregates, prim = mostly isolated primary particles.

The IINS results in Table 2 on the integrated normalized band intensities of the palladium hydride particles under 700 mbar hydrogen loading and at different steps of dehydrogenation by evacuation and, finally, thermal treatment, reveal clear numerical differences between the catalysts which can be traced back to their different morphologies (Fig. S1 and S2,† Table 1). From Table 2, there is a surprise, in that the largest area *i.e.* hydrogen content is for sample 4, Pd/CB300, the one calcined at 300 °C.

The non-porous Pd/CB catalyst shows the smallest primary particles which, however, are mostly grown to linear aggregates with enhanced hydrogen storage capability due to higher phase coherence, which is essential for the formation of β-palladium hydride. Therefore, the hydrogen value is higher (∼79) compared to the porous Pd/AC catalyst (∼60) which mostly shows isolated small primary particles. With strongly increasing primary particle size (Δ + 4.3 nm) from 2.4 to 6.7 nm due to the 300 °C thermal treatment, the overall hydrogen solubility increases (∼99) assisted by the presence of grown aggregates of also enlarged primary particles (Fig. S2†). Additional particle growth (Δ + 1 nm) together with completing carbo-thermal particle rearrangement and de-aggregation (400 °C), (Fig. 2 and S1†) finally causes some decrease of the hydrogen value for the polydisperse ensemble of now mostly isolated primary particles on the non-porous carbon support.

Unexpectedly, in the desorption experiments (Fig. 3, 4, S4, S5† and Table 2), it is observed that in spite of the small particle sizes and the well-known high diffusion coefficient^19^ of hydrogen in solid palladium it turned out that the complete removal of hydrogen from the catalyst particles by pumping at room temperature was very slow. This indicates significant hysteresis in the release of stored hydrogen from the finely divided catalyst particles. The presence of hysteresis has been studied by hydrogen absorption/desorption cycles in more compact palladium morphologies^19^,^20^ but recently was also extended to calibrated Pd nano-particles.^21^ The slow removal of hydrogen from the Pd/AC catalyst compared to Pd/CB (Table 2) can be explained by the high porosity of the AC support and the corresponding influence of pore diffusion and gas phase transport phenomena of molecular hydrogen after associative desorption of stored and chemisorbed hydrogen from the palladium particles.

**Fig. 5:**
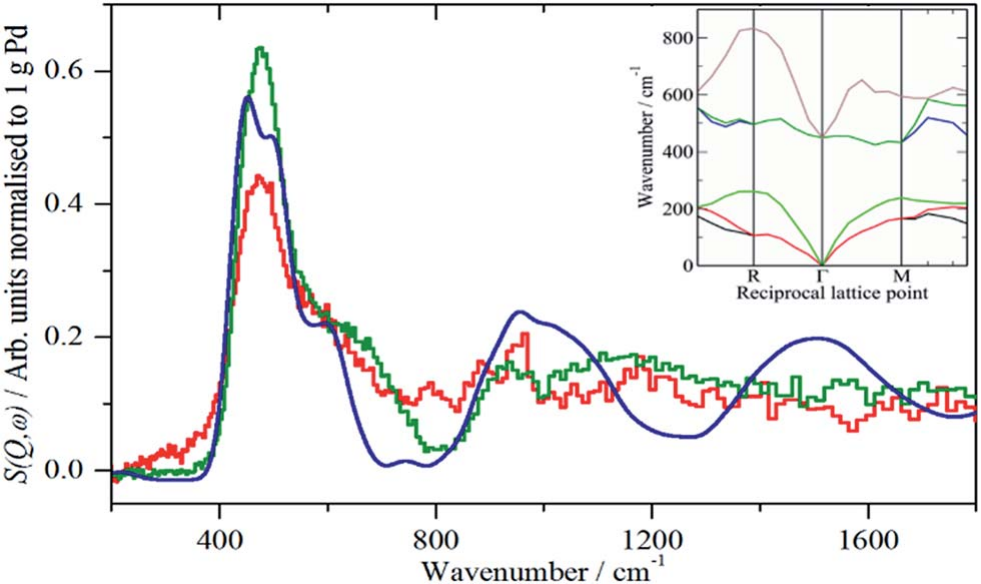
Comparison of the normalised IINS difference spectra of samples 2 (Pd/AC) and 4 (Pd/CB300) with that generated from a lattice dynamics calculation in the region of β-PdH. The inset shows the calculated dispersion curves.

A comparison of the spectra of samples 2 and 4 under H_2_ is shown in Fig. 5. It can be seen that the shape of the feature due to β-PdH is similar in both cases (samples 3 and 5 – Fig. S4 and S5† – are similar in shape to sample 4), although for the activated carbon support, sample 2, it is somewhat broader than for the samples supported on carbon black. All the samples show the asymmetric broadening typical of β-PdH. This is partly the result of strong dispersion in the optic modes, as seen experimentally^22^ for PdD_0.63_, and also calculated for β-PdH, Fig. 5 inset. It can be seen that the dispersion does not fully account for the width of the fundamental mode or its overtones. Since this is a calculation based on the harmonic approximation, it demonstrates that this is inadequate to describe β-PdH. Previous work^23^ has shown that the potential is strongly anharmonic along the [100]-direction, which would explain why the positions of the observed and calculated overtones differ. It has also been suggested^18^ that a Franck–Condon transition contributes to the shoulder at ∼600 cm^–1^.

Close inspection of the spectra of sample 2, Pd/AC, Fig. 3, shows weak features at 886, 961 and 1177 cm^–1^, which are more clearly seen in the ordinate expanded inset, Fig. 3b. These are assigned as C–H deformation modes of the carbon support and it appears that there has been hydrogasification by hydrogen spillover. Comparison with other recent IINS results show these resemble H1 and H2 sites, isolated and vicinal hydrogen at the edges of the basic structural units.^14^ Very surprisingly, this appears to be reversible or unstable: as the catalyst is dehydrogenated down from 700 mbar H_2_, the bands diminish, until they are of the same intensity as the pure support in the sample evacuated at 200 °C overnight. This only occurs for the partly disordered activated carbon support which has, comparatively, enhanced surface-ruggedness (TEM, Fig. 1) and surface polarity (XPS), and, therefore, reactivity in the formation of some partly volatile lower molecular surface species. There is no evidence for this occurring on the more graphitic carbon black support.

**Fig. 6:**
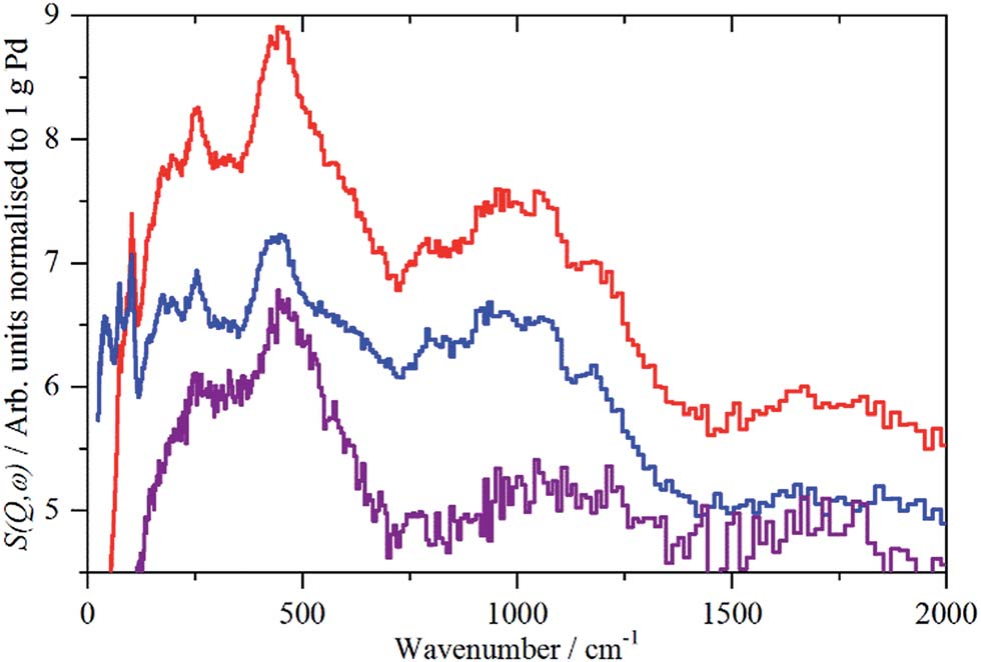
IINS spectra of Pd(1.045%)Au(0.43%)/SiO_2_. From top to bottom: catalyst under 700 mbar H_2_; after evacuation at 200 °C; difference spectrum (2× ordinate expanded).

For sample 1, Pd(1.045%)Au(0.43%)/SiO_2_, there was only sufficient time to measure the sample under 700 mbar H_2_ and after evacuation at 200 °C overnight, without additional intermediate steps of hydrogen desorption. The spectra of the catalyst under hydrogen and after evacuation are very similar and this is confirmed by the difference spectrum, Fig. 6. This consists of a peak at 480 cm^–1^, assigned as β-PdH, and a feature centred at ∼250 cm^–1^. The apparent normalized integrated area is very large, Table 2, twice that of the Pd/C samples. This value is not plausible, as alloying Pd with other metals can drastically reduce the hydrogen capacity of palladium.7b,c It is likely that there are contributions from other species, *e.g.* OH generated by spillover, that are wrongly included in the area.

The assignment of the ∼250 cm^–1^ feature is uncertain. Au is reported^24^ to form on-top surface hydrides, although this is controversial (see discussion in ref. 24b), if so, these could be the associated bending modes, although the transition energy is very low. It could also be the result of changes in the silica hydroxyls on evacuation.

### Surface and subsurface hydrogen

While the state of the hydrogen in β-PdH has been comprehensively studied,^1^,6e,^18^,^19^,^23^,^25^ the nature of the hydrogen at the surface has been almost completely neglected: we are unaware of any experimental studies in this area. There is also a surprising dearth of computational studies, the only one^26^ did not investigate the vibrational spectra.

**Fig. 7:**
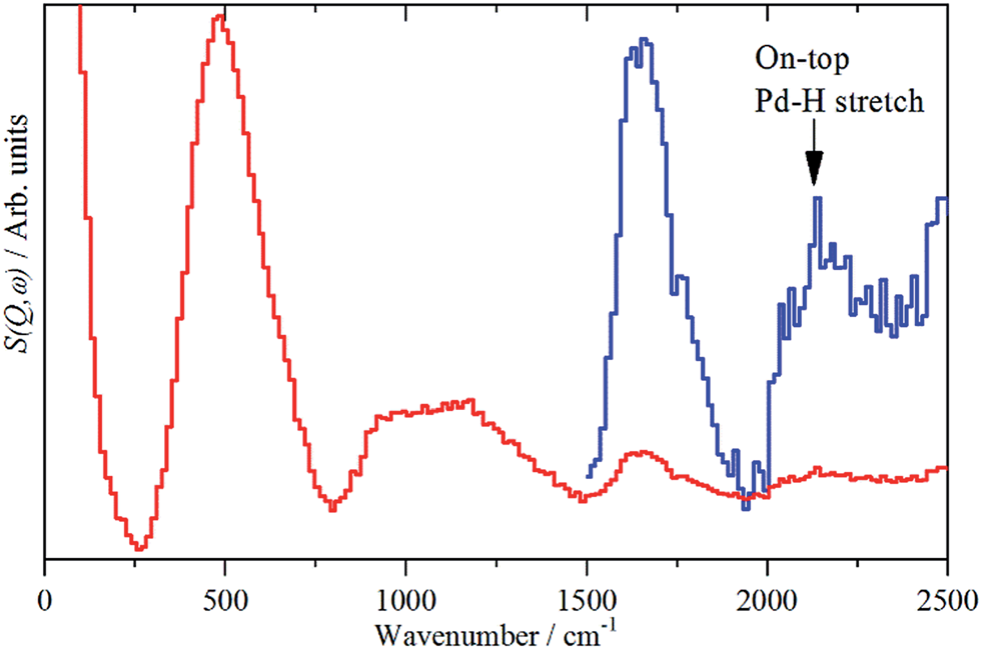
IINS difference spectrum of sample 6, (Pd/CB2), ([catalyst + 700 mbar H_2_] – [dehydrogenated catalyst]). The blue trace is a ×10 ordinate expansion of the 1500–2500 cm^–1^ region.

The sensitivity to hydrogen and the transparency of metals to neutrons means that IINS is the technique of choice to investigate hydrogen-in-metal systems.^27^ Almost all of these studies have been conducted with TOSCA or similar instruments, which are optimal below 1600 cm^–1^. The use of a different type of IINS spectrometer (‘direct geometry’^28^) enables the region >1600 cm^–1^ to be accessed and, in particular, has allowed observation of the Pt–H stretch of hydrogen in the on-top position of a platinum fuel cell catalyst.^29^Fig. 7 shows the difference spectrum of sample 6 (20% Pd on very low H-content carbon black, Table S1†) recorded with the direct geometry IINS spectrometer MERLIN (see ESI†). A spectrum recorded on a different instrument (MAPS) is shown in Fig. S6.† In addition to the β-PdH 0 → 1, 0 → 2 and 0 → 3 modes at 485, 1100 and 1640 cm^–1^ in both spectra a weak peak is evident at 2150 cm^–1^, this is assigned as the on-top Pd–H stretch mode. An alternative assignment to the 0 → 4 transition of β-PdH is ruled-out, as single crystal studies^23^ of β-PdH show no transitions at this energy.

Differences in the activity and selectivity of α- and β-phase hydride in the selective hydrogenation of ethyne are known^9^ and this may relate to the presence or not of the on-top surface site. We emphasise that while this paper reports the first observation of the on-top site, it is undoubtedly present under conditions where H_2_ gas is present, *i.e.* the β-PdH phase. Thus it is not new, it has always been present in catalytic hydrogenations by β-PdH, it has just not been recognised until now.

We have attempted to detect the bending modes of the on-top species by measuring the spectrum in the presence of H_2_ gas, then removing the H_2_ at 77 K and re-measuring the spectrum. In the case of platinum, the on-top species is only present with H_2_ present,^30^ so our expectation was that the same would hold for Pd. This is not the case and the spectra with and without H_2_ were the same.

Since both, the β-PdH signal and that of the on-top site at 2150 cm^–1^ are available from one single spectrum, a rough estimate of the ratio of hydrogen stored inside and located on the surface, of the supported palladium particles becomes possible. This gives a ratio of surface : bulk-hydrogen of 1 : 8, note that this includes an unknown contribution from hydrogen in high coordination sites at the surface, so is an underestimate of the relative populations. An alternative estimate can be obtained from the average particle size. The calculation yields a ratio of 1: 2.8. If the H atoms at the surface in high coordination sites are excluded, then the ratio becomes 1 : 2.1. (The details of both estimates are given in the ESI†).

The differences between H-surface : H-bulk from a model 1: 2.8 (2.1) and the experimental IINS result 1: 8 can be explained by the shape of the particles as shown in Fig. S1 and S2,† left. The presence of larger linear aggregates consisting of *ca*. 2.4 nm sized primary particles provide sufficient phase coherence for hydrogen storage (Table 2, Pd/CB *vs.* Pd/AC).

**Fig. 8:**
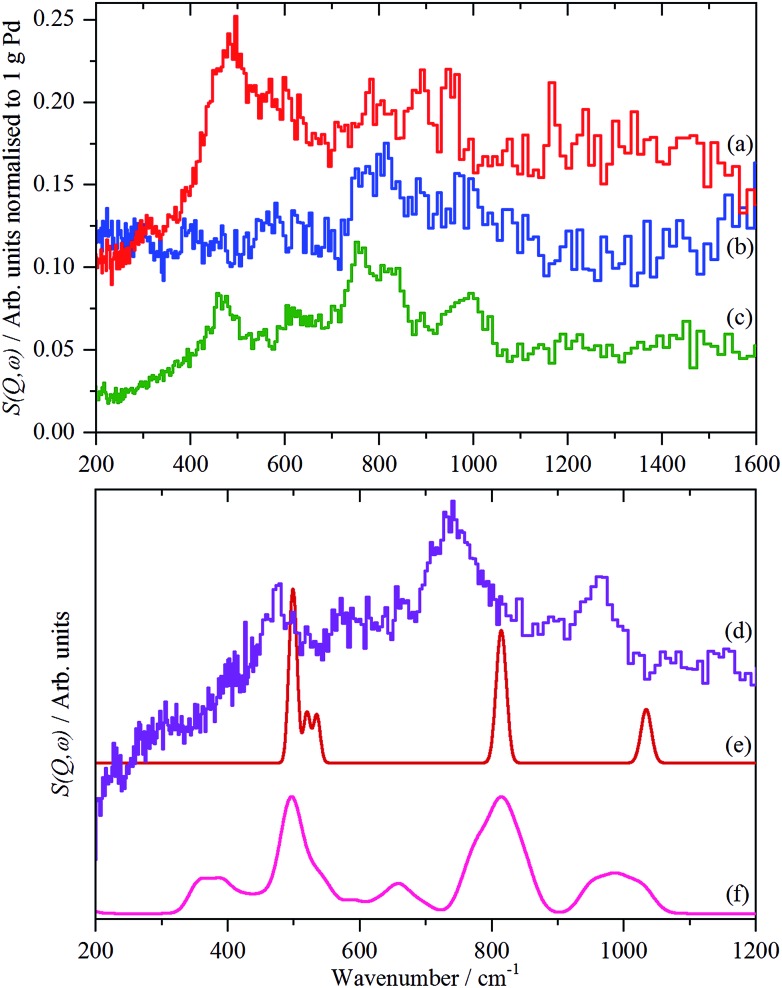
Normalized IINS difference spectrum of catalyst samples 2 (a), 3 (b) and 4 (c) in the region of β-PdH after dehydrogenation (b) is 2× ordinate expanded relative to (a) and (c). IINS spectrum of: (d) palladium black after dehydrogenation at 100 °C and compared to that calculated from the model shown in Fig. 9, (e) *Γ*-point in the Brillouin zone only and (f) including the complete Brillouin zone.

The spectra of the samples after evacuation at 200 °C overnight are shown in Fig. 8 and exhibit a distinct difference between the supports. For the activated carbon supported catalyst sample 2, the hydrogen residue is predominantly β-PdH (peak at 480 cm^–1^), a shoulder at ∼560 cm^–1^ is probably α-PdH.^31^ The same pattern is also seen in the palladium black sample after dehydrogenation, Fig. S7.† In contrast, the carbon black supported samples show weak bands at 470, 760/820 and 980 cm^–1^, the first of these is not seen on sample 3, suggesting that it is not associated with the other modes.

The spectrum of sample 4 is very similar to that of palladium black after extensive dehydrogenation, Fig. 8d, which has also been observed by others previously on this material.^32^ The spectrum was assigned32*c* as sub-surface hydrogen (470 cm^–1^) and the asymmetric and symmetric Pd–H stretch modes of hydrogen in a threefold surface site (760/820 and 980 cm^–1^, respectively) on (111) facets. Fig. 8b shows that the surface threefold sites are occupied before the sub-surface sites, Fig. 8c.

**Fig. 9:**
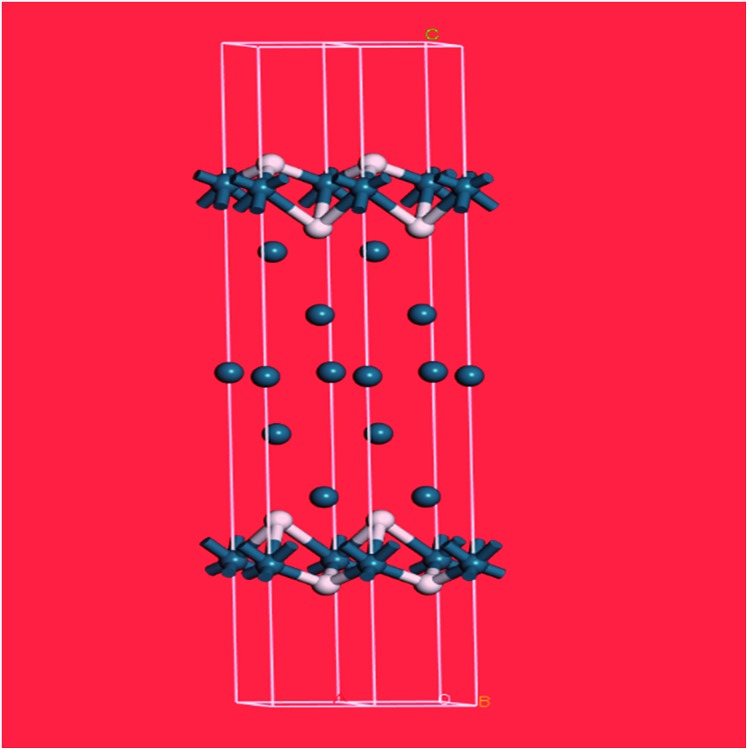
Model of hydrogen on Pd(111) at low coverage: the threefold and a sub-surface site are occupied. Two unit cells are shown. Pd = dark blue, H = white.

The splitting of the degenerate asymmetric stretch was considered to be due to mode dispersion indicative of strong lateral interactions. The assignment to a threefold site is consistent with HREELS spectra of the H/Pd(111) system.^33^Fig. 9 shows a model for the low coverage state consisting of hydrogen in a threefold surface site on Pd(111) and a subsurface site. The calculated spectrum at the *Γ*-point in the Brillouin zone is shown in Fig. 8e and that including the complete Brillouin zone in Fig. 8f. It can be seen that the agreement is reasonable. The subsurface species (bands at ∼500 cm^–1^) is calculated to be too strong, this reflects that the model assumes full occupation, which is probably not correct. The modes of the threefold site (bands at 815 and 1035 cm^–1^) are slightly overestimated but show the correct 2 : 1 relative intensity expected for the asymmetric and symmetric stretch, respectively. The shape of the bands is much better matched by the inclusion of dispersion confirming the earlier suggestion.32*c* The calculated dispersion curves, Fig. S8,† show that there is significant interaction between neighbouring hydrogen atoms, as also shown by isotope dilution measurements on this system.32*c*

The nature of the support is not only integral to the amount of hydrogen held by the catalyst, it also causes a marked difference in the rate of release of stored hydrogen from palladium. It is more difficult to fully dehydrogenate palladium on/in the porous activated carbon than on the non-porous carbon black. The type of support also results in differences in the form of the residual hydrogen: whether it is α- or β-hydride phase, subsurface or in the threefold surface site.

TEM and XPS demonstrate tremendous differences in the sp^2^ character, surface chemistry (functional groups) and free energy of the surface which can be further modified by additional thermal treatment as shown with the 300 °C and 400 °C Pd/CB catalysts. This illustrates the carbo-thermal reduction potential of a seemingly inert support. Differences in the support's porosity (activated carbon) and graphiticity (carbon black) and, therefore, size of the basic structural units (TEM) and electrical conductivity are direct influences on the spreading and the size and morphology of the precious metal entities over the support and this causes the differences between isolated primary particles and aggregates – during impregnation and also thermal after-treatment. This results in different proportions of adsorption sites at the surface and selvedge and on the overall hydrogen storage on the interstitial sites inside of the “bulk” of the supported Pd-nano-particles.

Differences in size and morphology (ratio of primary particles *vs.* aggregates formed by nanosized entities) affect the long range phase coherence. This influences the extent of the phase transition region and the relative proportions of hydrogen in more localized or delocalized palladium–hydrogen bonding. This conclusion is supported by the observation of hysteresis effects in hydrogen desorption (Table 2).

**Fig. 10:**
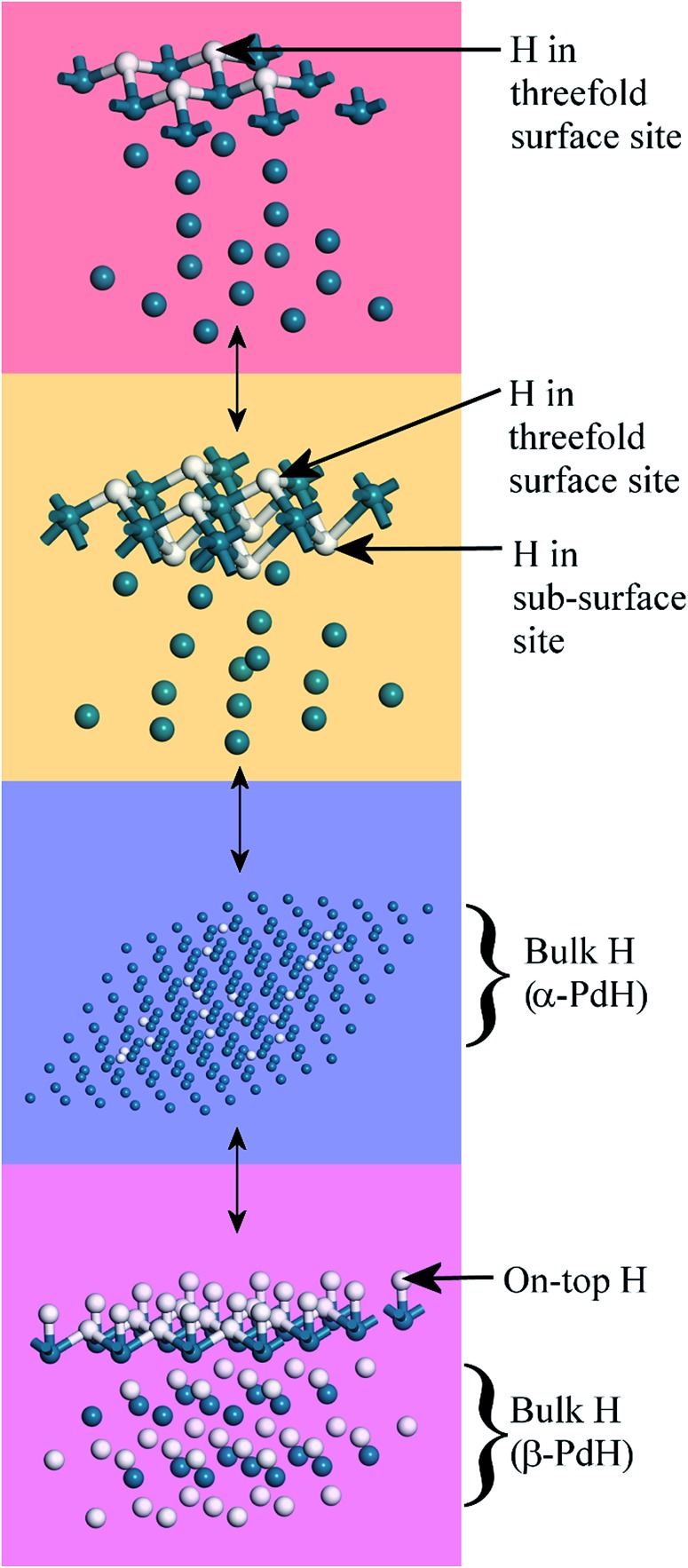
Summary of the evolution of hydrogen on palladium. Top to bottom: the threefold sites are initially occupied, with subsequent occupation of the threefold and subsurface sites. This leads to α-PdH and finally to β-PdH with the on-top sites occupied. The sequence is fully reversible. Pd = dark blue, H = white.

Fig. 10 summarises the states of hydrogen on palladium as a function of hydrogen content: at the lowest hydrogen content only the surface threefold sites are occupied, the sub-surface site is then filled, migration of this into the bulk generates α-PdH and further hydrogen results in formation of β-PdH with the on-top sites occupied.

## Conclusions

The relative amounts of hydrogen retained by a range of supported palladium catalysts have been investigated by a combination of electron microscopy and spectroscopic techniques. Contrary to expectation, the hydrogen capacity is not determined solely by the metal particle size, but it is a complex interaction between the particle size and its state of aggregation, which is heavily influenced by the choice of support. The interplay of these two factors offers new opportunities to fine-tune the amount of hydrogen available in a catalytic process.

We have examined the nature of hydrogen adsorbed at the surface of β-PdH and have observed for the first time hydrogen in the on-top site. This underlines the importance of the hydrogen carrier function of palladium catalyst for the local hydrogen balance in catalyst operation.

As quoted in the Introduction: ‘in many cases a minimum concentration of dissolved hydrogen in the liquid in contact with the solid catalyst is needed’.^11^ This work has clearly demonstrated that the nature of the hydrogen present; on-top, surface or bulk, depends not only on the presence, or not, of hydrogen gas but is also critically dependent on the palladium morphology, which is largely determined by the support.

## Author contributions

P. W. A. conceived the project. J. A., E. G., T. P., D. W., K. M. and S. D. W. prepared and provided the samples and information. S. F. P. and P. W. A. carried out the neutron measurements with H. C. W. (MERLIN), S. K. C. (SANDALS) and M. J.-R. (IN1-Lagrange). S. F. P. and P. W. A. wrote the paper with input from all co-authors.

## Conflicts of interest

There are no conflicts to declare.

## Supplementary Material

Supplementary information
