# Supplementary material for: The effect of particle size, morphology and support on the formation of palladium hydride in commercial catalysts
Source: Chem Sci. 2018 Oct 15;10(2):480–9. doi: 10.1039/c8sc03766c (PMC6334263; doi:10.1039/c8sc03766c)
Supplement: Supplementary file 1 [file SC-010-C8SC03766C-s001.pdf]

Electronic Supplementary Material (ESI) for Chemical Science.
This journal is © The Royal Society of Chemistry 2018

# Electronic Supporting Information (ESI) for:

# The effect of particle size, morphology and support on the formation of palladium hydride in commercial catalysts

Stewart F. Parker*[a], Helen C. Walker,[a] Samantha K. Callear,[a] Elena Grünewald,[b] Tina Petzold,[b] Dorit Wolf,[b] Konrad Möbus,[b] Julian Adam,[b] Stefan D. Wieland,[b] Mónica Jiménez-Ruiz[c] and Peter W. Albers*[d]

[a]*ISIS Facility, STFC Rutherford Appleton Laboratory, Chilton, Didcot, OX11 0QX, UK*
[b]*Evonik Resource Efficiency GmbH, Rodenbacher Chaussee 4, D-63457 Hanau-Wolfgang, Germany*
[c]*Institut Laue-Langevin, 71 Avenue des Martyrs, CS 20156 38042 Grenoble Cedex 9, France*
[d]*Evonik Technology and Infrastructure GmbH, Rodenbacher Chaussee 4, D-63457 Hanau-Wolfgang, Germany*

Corresponding authors: peter.albers@evonik.com and stewart.parker@stfc.ac.uk

**Table of contents**

**Experimental Section** ... 3

Materials ... 3

Transmission electron microscopy (TEM) ... 3

Scanning electron microscopy (SEM) ... 4

X-ray photoelectron spectroscopy (XPS) ... 4

Inelastic incoherent neutron scattering (IINS) ... 4

Neutron diffraction ... 5

Computational studies ... 5

**Results and discussion** ... 6

Fig. S1 TEM images of the catalysts ... 6

Fig. S2 TEM: Palladium size distribution of the catalysts. ... 7

Fig. S3 SEM/EDX element mappings of sectioned and polished $SiO_2$ particles ... 8

Fig. S4 IINS spectra of sample 3 (Pd/CB) ... 9

Fig. S5 IINS spectra of sample 5 (Pd/CB400) ... 10

Table S2: Sample treatments and integrated hydrogen areas ... 11

Fig. S6 20%Pd/CB catalyst under 700 mbar $H_2$ showing the on-top hydrogen ... 12

Estimation of the relative proportion of the on-top site to that of bulk β-PdH ... 13

Fig. S7 Palladium black with residual hydrogen ... 14

Fig. S8 Dispersion curves of surface and sub-surface hydrogen on Pd(111) ... 15

**References** ... 16

## Experimental Section

### Materials

Porous and non-porous high purity carbon were used as support materials in this study: a powder type high surface area activated carbon (AC), (nitrogen surface area > 1000 $m^2/g$, steam activated, total pore volume > 1.3 ml $g^{-1}$, hydrogen content 3900 ppm H), and carbon black (CB) with enhanced $sp^2$ character as indicated by the low hydrogen content (295 ppm H). The hydrogen content of the carbon supports was determined by hot extraction using a LECO TCH600 instrument. The freshly impregnated 20% Pd/CB catalyst was additionally treated by carbo-thermal reduction at 300 ° and 400 °C in an argon atmosphere. In the literature, this treatment is described as a suitable means to produce fine particle distributions of colloidal entities at the surface of a non-porous carbon black support.[1] It also allows us to numerically probe the influence of temperature on controlled particle growth, the changes of average particle size and morphology, the impact on hydrogen absorption capacity and site occupation. In addition, a Pd(20%)/CB with a very low hydrogen content (60 ppm H) that has been previously described[2] and a shell-type Pd(1.04%)/Au(0.43) alloy catalyst supported on silica-pellets were also studied. The sample masses used in an individual IINS measurement were in the range 14 - 50 g, Table S1, so provided a representative, average sample of the catalysts, all of which were manufactured by Evonik Resource Efficiency GmbH.[3]

A 60.5 g sample of high purity palladium black (99,11%; BET surface $\geq 25$ $m^2$ $g^{-1}$; CAS No. 7440-05-3; Umicore Precious Metals Chemistry) was also investigated. Further details on this material are given elsewhere.[2]

**Table S1** Catalyst samples and amount probed in a single neutron experiment.

| **Sample No./catalyst** | **IINS sample weight / g** |
|---|---|
| Activated Carbon support background | 18.57 |
| Carbon Black support background | 14.34 |
| 1 1.04%Pd,0.43%Au/$SiO_2$ (PdAu) | 49.57 |
| 2 20% Pd on Activated Carbon (Pd/AC) | 27.08 |
| 3 20% Pd on Carbon Black (295 ppm H) (Pd/CB) | 32.27 |
| 4 as 3 calcined 300°/Ar (Pd/CB300) | 25.51 |
| 5 as 3 calcined 400°/Ar (Pd/CB400) | 28.77 |
| 6 20% Pd on Carbon Black (60 ppm H) (Pd/CB2) | 25.79 |

### Transmission Electron Microscopy (TEM)

A Hitachi H7500 and a Jeol 2010F field emission transmission electron microscope were operated at 100 and 200 keV acceleration voltage, respectively. For confirming the chemical identity of precious metal primary particles and aggregates and regarding alloying in the Pd/Au-catalyst, energy dispersive X-ray spot-analyses at the nanoscale (EDX) of the supported particles and the support particles were performed using a Noran SiLi detector with a 30 $mm^2$ crystal and a Noran System Six device. A catalyst sample was dispersed and transferred onto Holey Carbon Foil supported by a 200 mesh copper grid. For statistical evaluation of the primary particle sizes of the supported primary particles the I-TEM software of Soft Imaging Systems (SIS), Münster, Germany, was utilized. The average primary particle

sizes of the supported Pd-based catalyst particles were determined by statistical evaluation of 2000 particles in TEM images per catalyst sample (Table 2) and Fig. S1. The quality, stability and calibration of the TEM system were maintained by the use of the Magical No. 641 standard (Norrox Scientific Ltd., Beaver Pond, Ontario, Canada).

**Scanning Electron Microscopy (SEM)**

A Jeol 7600F field emission instrument with EDX detector (150 mm$^2$, Oxford Aztec Advanced System) was used.

**X-ray Photoelectron Spectroscopy (XPS)**

A Thermo Fisher 250Xi instrument was operated using mono-chromatised Al Kα radiation, 20 eV pass energy A catalyst sample was introduced into a differentially pumped pre-chamber as a loose powder. Integral XPS spectra of an area of 1 mm$^2$ size were recorded.

**Inelastic Incoherent Neutron Scattering (IINS)**

Each catalyst was sealed into a thin-walled (0.5 mm) stainless steel (1.4571) can which was closed by a top-flange with a stainless steel pipe and a welded bellows valve (Nupro) *via* a OFHC-copper (oxygen-free high conductivity) gasket. A sealed can containing macroscopic amounts of catalyst (Table 1) was evacuated using a turbo-molecular pump which was backed by a dual stage rotary pump with a zeolite trap to avoid back-diffusion of oil and other potential molecular contaminants. In order to remove surface-hydroxides, traces of adsorbed water and sub-stoichiometric surface-oxides, each catalyst was subjected to slow and careful cycles of hydrogenation (99.999% hydrogen, Linde) and dehydrogenation at room temperature to avoid fast local heating due to a spontaneous excessive release of the heat of the dissociative absorption of hydrogen in the palladium in fast-step dosing. No heating during degassing of a hydrogenated catalyst by pumping down a sample can was performed, in order to avoid particle growth induced by heat under the presence of residual pressure of hydrogen and further reactions. The hydrogen uptake of the supported palladium was monitored by capacitive pressure transducers (Baratron). After 5 cycles at room temperature and 24 h of evacuation with a turbomolecular pump each sample was topped to 700 mbar hydrogen sorption equilibrium pressure and measured with IINS.

IINS spectra were recorded on TOSCA,[4,5] a high resolution indirect geometry spectrometer. Spectra of the surface occupation in on-top site were recorded with the MAPS[4,6] and MERLIN,[7] direct geometry chopper (all at the ISIS Facility, STFC Rutherford Appleton Laboratory, Chilton UK). Neutron spectra on the hydrogenated palladium black reference sample were taken at IN1-Lagrange, a high resolution beryllium filter spectrometer using the Cu(220) monochromator[8,9] (ILL, Institute Max von Laue/Paul Langevin, Grenoble, France). The different characteristics of the instruments are discussed in detail elsewhere.[10] For the present work, the relevant properties are that TOSCA and IN1-Lagrange provide excellent spectra in the 0 – 1600 cm$^{-1}$ region, while MAPS and MERLIN provide access to the 1600 – 4000 cm$^{-1}$ region. MAPS provides good resolution and modest sensitivity while MERLIN provides modest resolution and excellent sensitivity.

All of the TOSCA spectra shown are normalised to 1 g Pd (or equivalently, 4 g carbon), allowing relative intensities to be compared. For the Pd/C catalysts, the hydrogen was pumped off in several steps and the sequential decomposition of the hydride phase measured by means of IINS. Difference spectra were generated by subtraction of either the sample after prolonged evacuation at 200 °C or the appropriate reference carbon. Finally, residual hydrogen was removed by pumping at 200 °C for recording background runs. For the Pd/Au/$SiO_2$ catalyst only time to record the spectrum under hydrogen and the 200 °C degassing run was available.

**Neutron Diffraction**

The catalyst was prepared as described in the previous section. It was then transferred to a flow-through TiZr cell in an argon-filled glovebox. The total scattering neutron diffraction pattern was then measured at room temperature using the SANDALS[11] diffractometer at the ISIS Pulsed Neutron and Muon Facility[4] (Chilton, UK). The sample was then dosed *in situ* with hydrogen. The sample was then measured again. Data reduction was carried out with the Gudrun package.[12]

**Computational Studies**

Periodic density functional theory calculations (periodic-DFT) calculations were carried out using a plane wave basis-set and pseudopotentials as implemented in the CASTEP code.[13,14] The generalised gradient approximation (GGA) Perdew-Burke-Ernzerhof (PBE) functional was used in conjunction with optimised norm-conserving pseudopotentials with a plane-wave cut-off energy of 1050 eV. 15×15×15 (120 k-points) and 16×16×1 (30 k-points) Monkhorst-Pack k-point grids were used for the bulk and the surface species respectively. Phonon modes were calculated using density-functional perturbation-theory.[15] As a prerequisite to any lattice dynamics calculation a full geometry optimization of the internal atomic co-ordinates was performed, the residual forces were |0.0025| eV $Å^{-1}$. The output of the phonon calculation includes the atomic displacements of the atoms in the mode, which was used to generate the INS spectra with ACLIMAX.[16] The transition energies have *not* been scaled.

**Results and discussion**

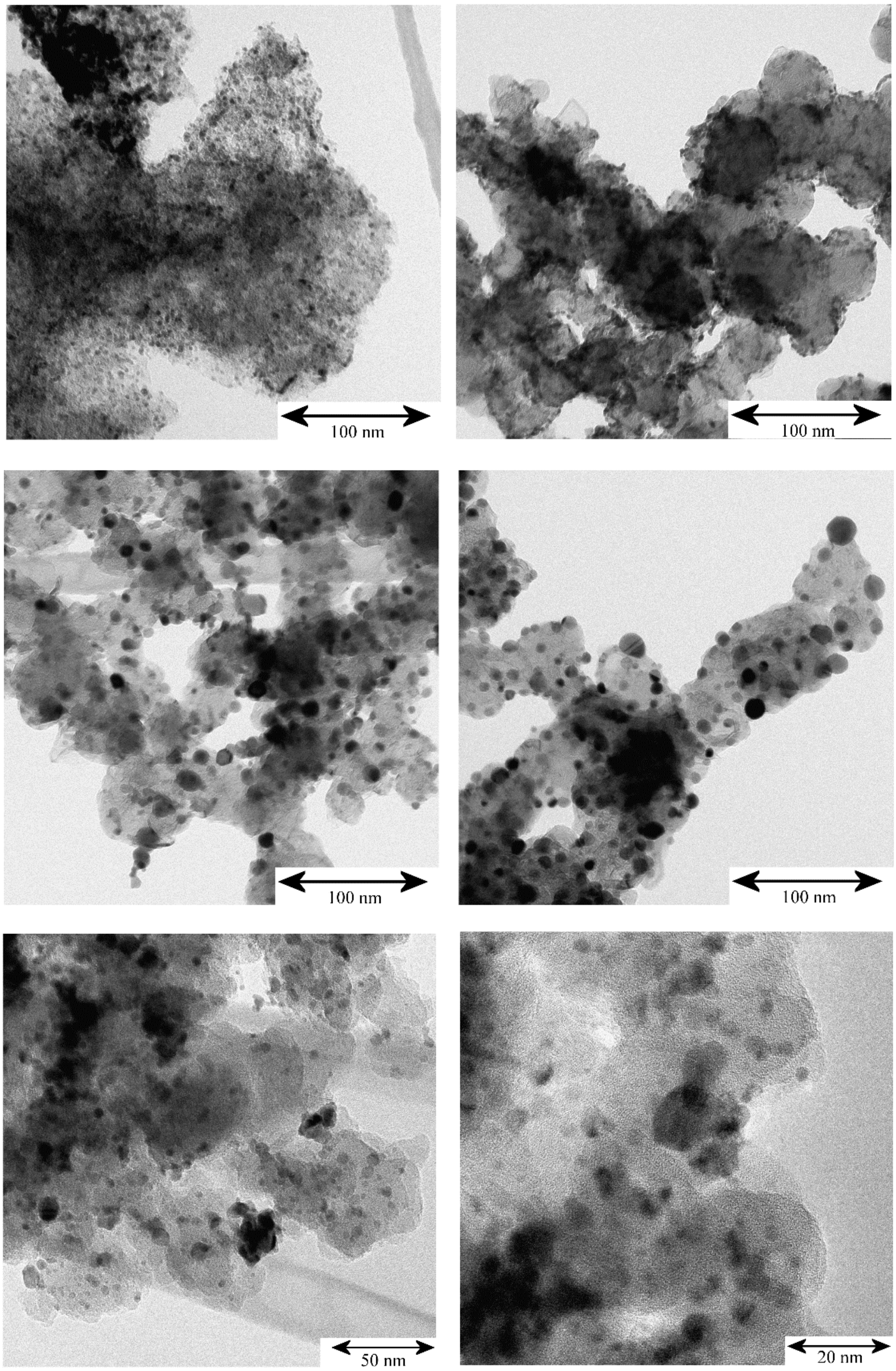


**Fig. S1** TEM images. Top: left, Pd/AC (mostly isolated primary particles on/in the porous high surface area AC support), right, Pd/CB (mostly linear aggregate chains and branched aggregates, some agglomerates at the surfaces); middle: Pd/CB300 °C, Pd/CB400 °C (mostly isolated primary particles of enlarged size (Table 1) and only few aggregates/agglomerates left), scale bar 100 nm; bottom: Pd/Au alloy catalyst on $SiO_2$ pellet support (mostly isolated primary particles of larger size).

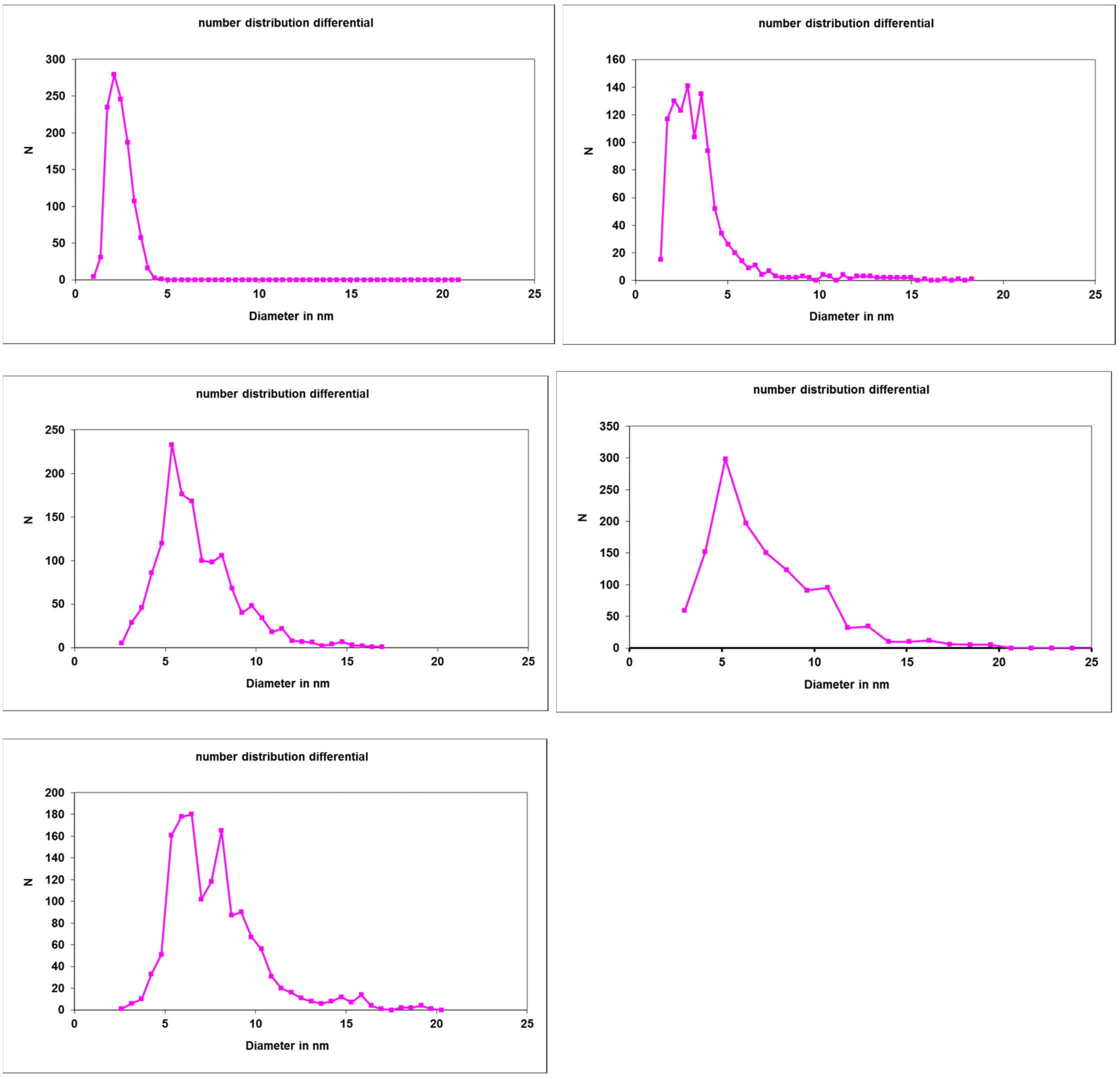


**Fig. S2** TEM: Size distribution determined by evaluation of *ca*. 2000 isolated as well as aggregated primary particles per sample. Top to bottom, left: Pd/CB, Pd/CB300 °C, Pd/CB400 °C, right: Pd/AC, $PdAu/SiO_2$

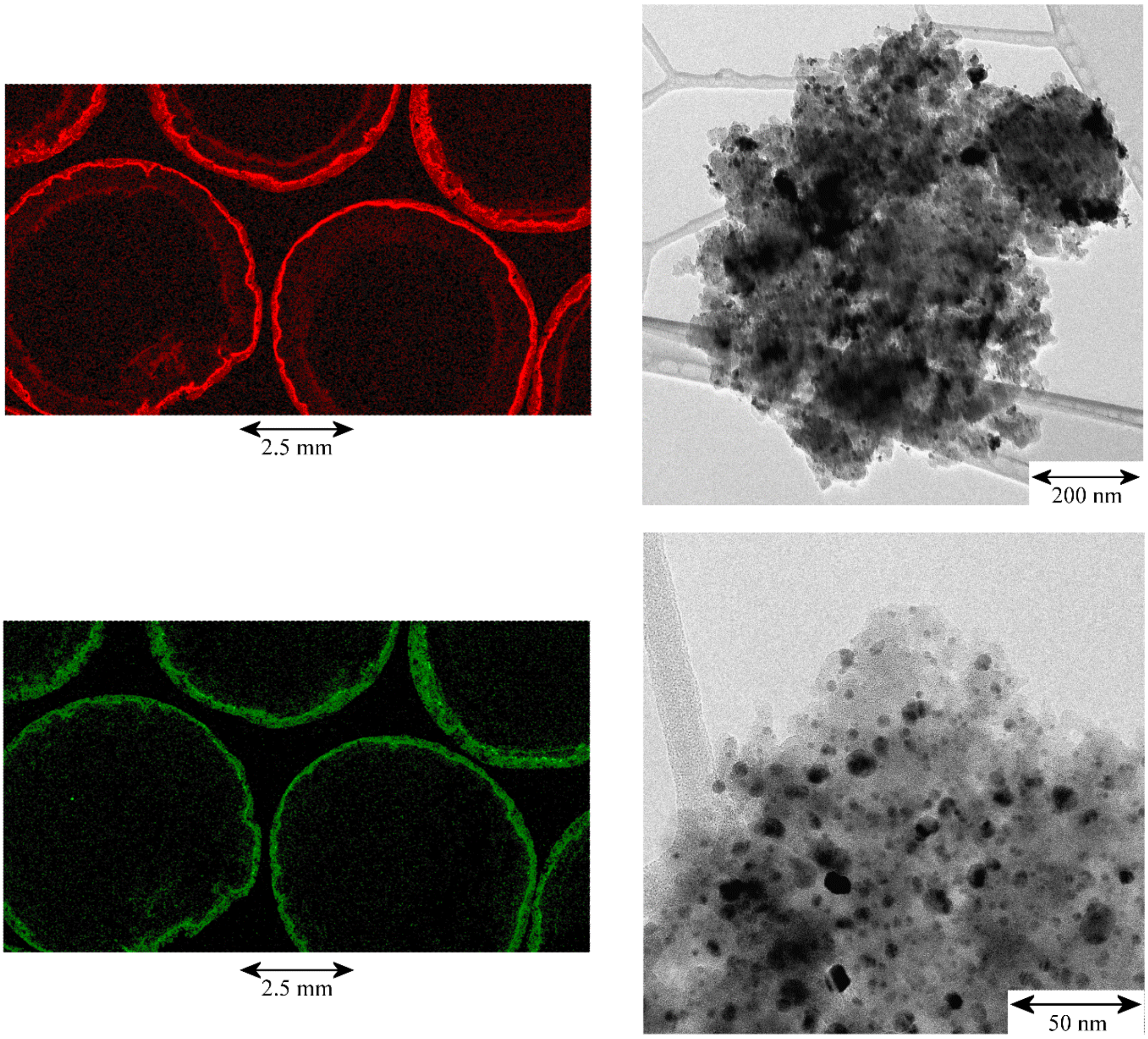


**Fig. S3** Left: SEM/EDX element mappings of sectioned and polished $SiO_2$ pellet particles illustrating the close local correlation of Pd (top) and Au (bottom) at the outer perimeter of the shell-type catalyst at the macroscale. Right: TEM of abraded particles from the outer surface for nano-spot EDX analyses which confirm that mostly Pd/Au alloy particles are present.

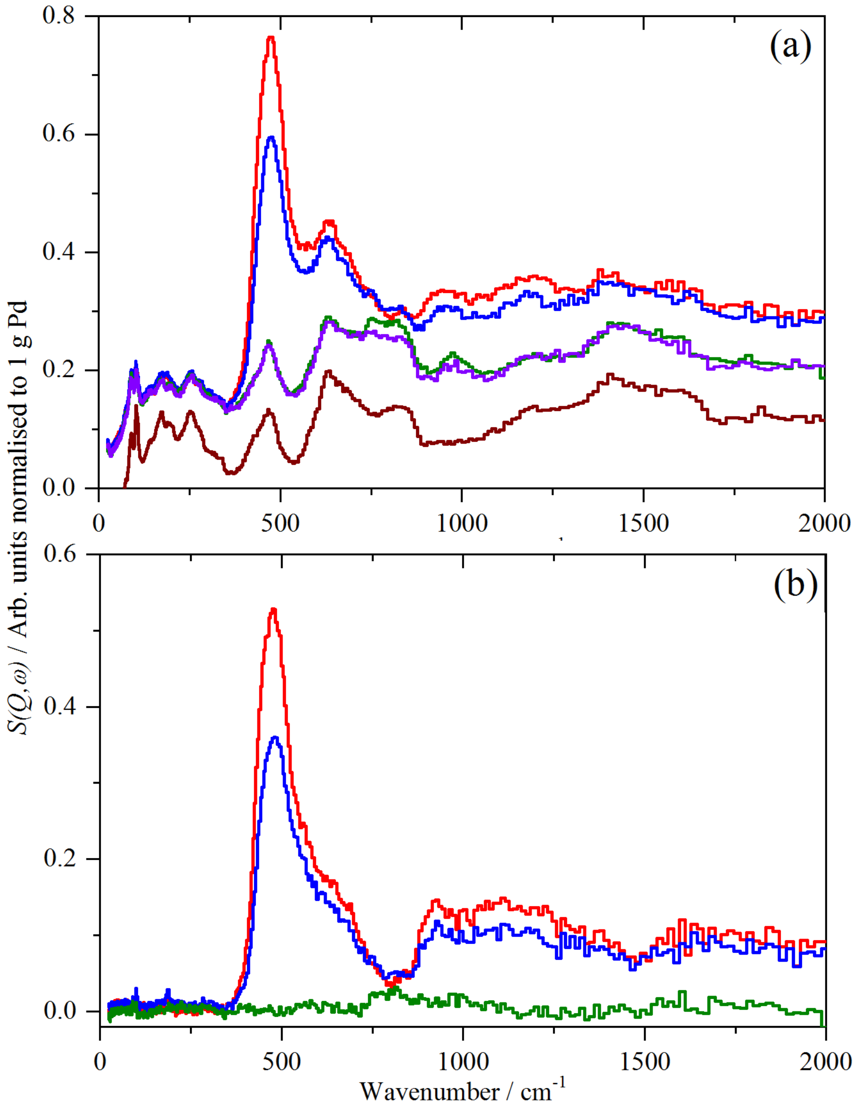


**Fig. S4** IINS spectra of sample 3 (Pd/CB): (a): normalised spectra as recorded, top-to-bottom: under 700 mbar $H_2$, evacuated to 14.5 mbar $H_2$, evacuated to 0.1 mbar $H_2$, evacuated at 200 °C overnight and the carbon black support. (b) Difference spectra after subtraction of the sample evacuated at 200 °C overnight, top-to-bottom: under 700 mbar $H_2$, evacuated to 14.5 mbar $H_2$, evacuated to 0.1 mbar $H_2$.

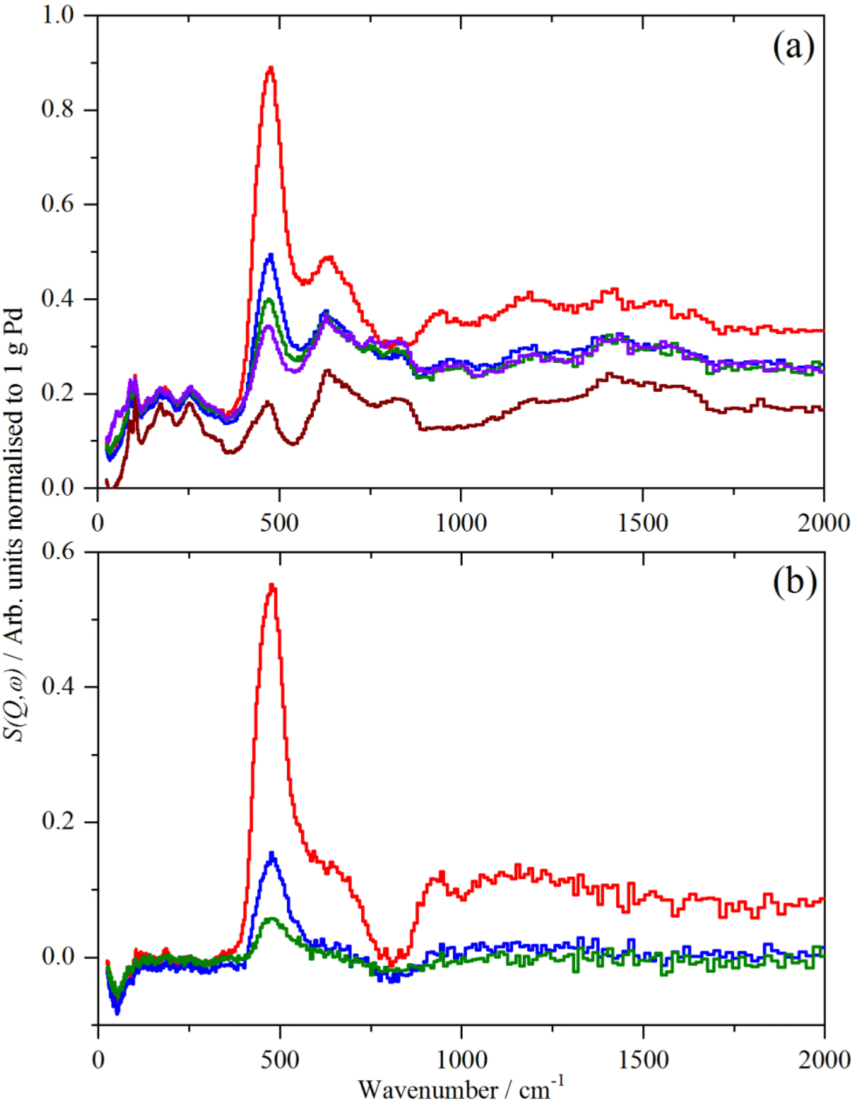


**Fig. S5** IINS spectra of sample 5 (Pd/CB400): (a): normalised spectra as recorded, top-to-bottom: under 700 mbar $H_2$, evacuated to 10 mbar $H_2$, evacuated to 5 mbar $H_2$, evacuated at 200 °C overnight and the carbon black support. (b) Difference spectra after subtraction of the sample evacuated at 200 °C overnight, top-to-bottom: under 700 mbar $H_2$, evacuated to 10 mbar $H_2$, evacuated to 5 mbar $H_2$.

**Table S2:** Sample treatments and integrated hydrogen areas (arbitrary units, normalised to 1 g Pd).

| Sample | 700 mbar $H_2$ | | 1st evac | | | 2nd evac | | | 3rd evac | |
|---|---|---|---|---|---|---|---|---|---|---|
| | Area | Limits / $cm^{-1}$ | Area | Limits / $cm^{-1}$ | P / mbar | Area | Limits / $cm^{-1}$ | P / mbar | Area | Limits / $cm^{-1}$ |
| 1 Pd/Au | 180.00 | From curvefit | | | | | | | | |
| 2 | 41.33 | 400-800 | 23.41 | 400-700 | 9 | 20.68 | 400-700 | 3 | 11.4 | 400-700 |
| 2 | 59.87 | 350-800 | 32.97 | 350-800 | 9 | 29.66 | 350-800 | 3 | 12.2 8.05 | 350-800 |
| 3 | 78.87 | 350-800 | 55.14 | 350-800 | 14.5 | 6.84 | 600-1200 | 0.1 | | |
| 4 | 99.00 | 350-800 | 74.66 | 350-800 | 13.3 | 18.68 | 350-1065 | 3 | | |
| 5 | 67.27 | 400-800 | 13.54 | 400-600 | 10 | 4.93 | 400-600 | 5 | 9.98 | 415-670 |
| 5 | 77.97 | 350-800 | 23.15 | 350-800 | 10 | 9.06 | 350-800 | 5 | | |

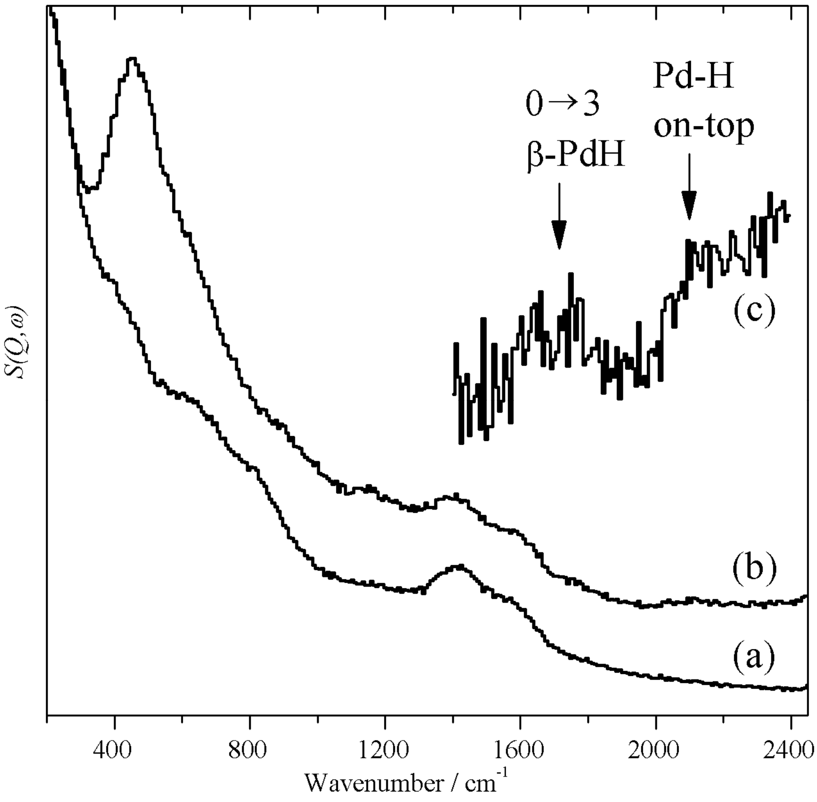


**Fig. S6** 20%Pd/CB catalyst under 700 mbar $H_2$ equilibrium pressure recorded with the direct geometry spectrometer MAPS; (a) background of the clean catalyst, (b) measured under 700 mbar hydrogen equilibrium pressure and (c) ×5 ordinate expansion of (b) in the 1400 – 2400 $cm^{-1}$ region.

**Estimation of the relative proportion of the on-top site to that of bulk β-PdH**

Since both, the β-PdH signal and that of the on-top site at 2150 $cm^{-1}$ are available from one single spectrum, (Fig. 7, main text), a rough estimate of the ratio of hydrogen stored inside the nanoparticle as β-PdH and located on the on-top sites of the supported palladium particles becomes possible. A total neutron scattering study of this catalyst showed that at room temperature the sample was actually β-$PdH_{0.7}$, as often found for nanoparticulate palladium.[17] Integration of the IINS area of the 0 → 1 transition of β-$PdH_{0.7}$ centred at 485 $cm^{-1}$ and the area of the on-top Pd-H-stretch region gave 22248 and 351 (arb. units), respectively. The β-$PdH_{0.7}$ area also includes the bending modes of the on-top species. Their contribution can be estimated as:
(area of on-top) × 2 (since two bending modes) × 4 (ratio of amplitude of motion of stretch to bend, assuming the harmonic approximation) = 2808
Hence ratio of surface: bulk-hydrogen = 2808 : 22248 = 1 : 8 (note that this includes an unknown contribution from hydrogen in high coordination sites at the surface, so is an underestimate of the relative populations).

The relative populations can also be estimated from the particle size. Assuming, as a first approximation, the primary particle size of the palladium is a cube of PdH with a length of 2.28 nm on each edge, has a diagonal length of 3.78 nm, which is in the right size regime (Table 1). Each edge has 6 Pd atoms (and 6 H atoms), hence 36 Pd per side, hence 6 x 36 = 216 surface Pd atoms. The cube contains a total of 863 Pd atoms + 863 H atoms, (note that 216 of these are at the surface in high coordination sites). If we assume the actual stoichiometry is *ca.* $PdH_{0.7}$[10] at the 700 mbar hydrogen loading pressure, it means there are 604 H atoms present. Assuming each surface Pd atom is capped by an on-top H, this gives a surface-to-bulk ratio of 216 : 604 *i.e.* 1 : 2.8. If we exclude the H atoms at the surface in high coordination sites, then the ratio is 216 : (604 – 216 x 0.7) = 216 : 453 *i.e.*1 : 2.1.

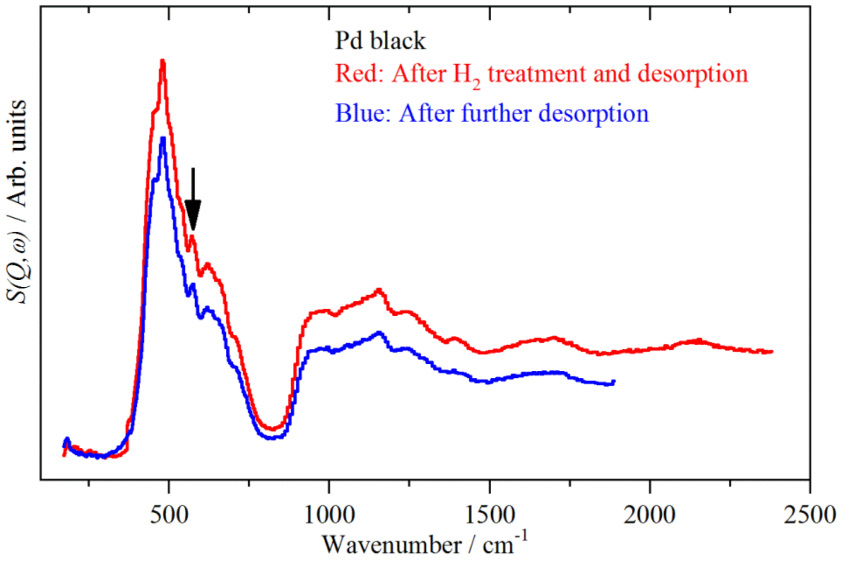


**Fig. S7** Palladium black with residual hydrogen loading of β-PdH after two dehydrogenation steps. The main peak at 485 $cm^{-1}$ and the smaller one at 640 $cm^{-1}$ are assigned to β-PdH,[17e] the overtone is centred at 1050 $cm^{-1}$. The weak peak at 574 $cm^{-1}$ (arrowed) is assigned to α-PdH.[18] Spectra recorded using IN1-Lagrange[9] at the ILL[8] (the spectrum of the original black before hydrogenation is shown elsewhere.[19]

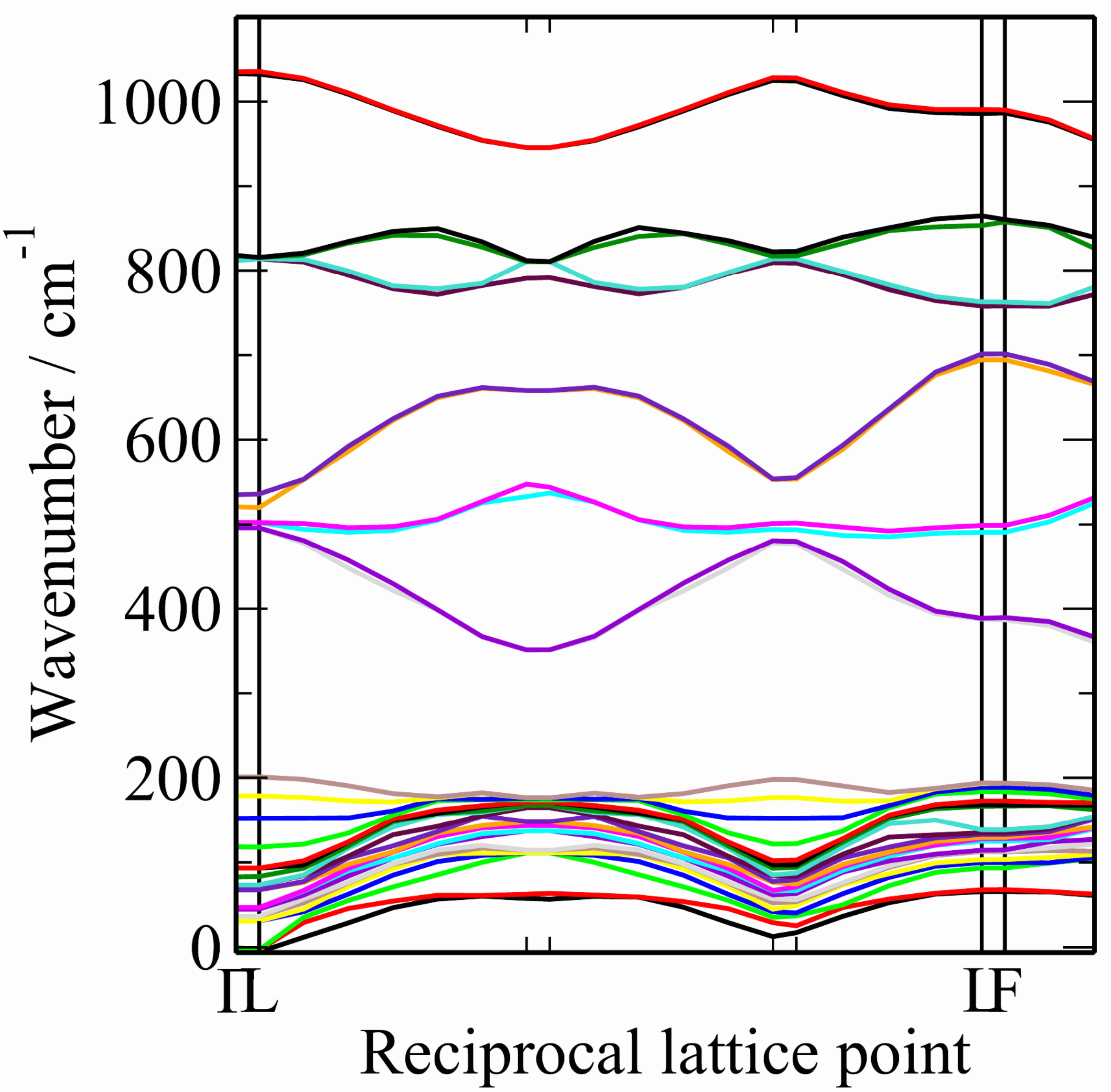


**Fig. S8** Dispersion curves of surface and sub-surface hydrogen on Pd(111) calculated from the model shown in Fig. 9 of the main text. Note that the modes are real across the entire Brillouin zone.

## References


1 A. Stassi, I. Gatto, V. Baglio and A.S. Aricò, *Int. J. Hydrogen Energy*, 2014, **39**, 21581-21587.

2 S. F. Parker, D. Adroja, M. Jiménez-Ruiz, M. Tischer, K. Möbus, S. D. Wieland, P. W. Albers, *Phys. Chem. Chem. Phys.*, 2016, **18**, 17196-17201.

3 (a) http://catalysts.evonik.com/product/catalysts/en/catalyst-brands/ ;
(b) http://catalysts.evonik.com/product/catalysts/en/downloads/ .

4 https://www.isis.stfc.ac.uk/Pages/Instruments.aspx

5 S. F Parker, F. Fernandez-Alonso, A. J Ramirez-Cuesta, J. Tomkinson, S. Rudic, R. S Pinna, G Gorini and J. Fernández Castañon· *J. Phys.: Conf. Ser.*, 2014, **554**, 012003.

6 S.F. Parker, D. Lennon and P. W. Albers, *Applied Spectroscopy*, 2011, 65, 1325-1341.

7 R. I. Bewley, R. S. Eccleston, K. A. McEwen, S. M. Hayden, M. T. Dove, S. M. Bennington, J. R. Treadgold and R. L. S. Coleman, *Physica B*, 2006, **385-386**, 1029-1031.

8 https://www.ill.eu/users/instruments/

9 M. Jiménez-Ruiz, A. Ivanov and S. Fuard, *J. Phys.: Conf. Ser.*, 2014, **554**, 01244.

10 P. C. H. Mitchell, S. F. Parker, A. J. Ramirez-Cuesta and J. Tomkinson, *Vibrational spectroscopy with neutrons, with applications in chemistry, biology, materials science and catalysis*, World Scientific, Singapore, 2005, ch. 3.

11 A. C. Hannon, W. S. Howells and A. K. Soper, *Inst. Phys. Conf. Series* 1990, **107**, 193.

12 http://www.isis.stfc.ac.uk/instruments/sandals/data-analysis/gudrun8864.html.

13 S. J. Clark, M. D. Segall, C. J. Pickard, P. J. Hasnip, M. J. Probert, K. Refson and M. C. Payne. *Z. Krist.*, 2005, **220**, 567-570.

14 K. Refson, P. R. Tulip and S. J. Clark, *Phys. Rev. B*, 2006, **73**, 155114.

15 V. Milman, A. Perlov, K. Refson, S. J. Clark, J. Gavartin and B. Winkler, *J. Phys.: Condens. Matter*, 2009, **21**, 485404.

16 A. J. Ramirez-Cuesta, *Comput. Phys. Commun.*, 2004, **157**, 226-238.

17 (*a*) A. Pundt, M. Suleiman, C. Bähtz, M. T. Reetz, R. Kirchheim and N. M. Jisrawi, *Mat. Sci. Eng. B*, 2004, **108**, 19-23; (*b*) A. Pundt, C. Sachs, M. Winter, M. T. Reetz, D. Frisch and R. Kirchheim, *J. Alloys Compounds* 1999, **293-295**, 480-483; (*c*) C. Sachs, A. Pundt, R. Kirchheim, M. Winter, M. T. Reetz and D. Frisch, *Phys. Rev. B*, 2001, **64**, 075408; (*d*) M. Suleiman, N. M. Jisrawi, O. Dankert, M. T. Reetz, C. Bähtz and R. Kirchheim, *J. Alloys Compounds*, 2003, **356/357**, 644-648; (*e*) P. Albers, M. Poniatowski, S. F. Parker and D. K. Ross, *J. Phys.: Condens. Matter*, 2000, **12**, 4451-4463.

18 J. J. Rush, J. M. Rowe and D. Richter, *Z. Phys. B Cond. Matter*, 1984, **55**, 283-286.

19 S. F. Parker, D. Adroja, M. Jiménez-Ruiz, M. Tischer, K. Möbus, S. D. Wieland and P. W. Albers, *Phys. Chem. Chem. Phys.*, 2016, **18**, 17196-17201.
